# Novel eIF4A1 inhibitors with anti‐tumor activity in lymphoma

**DOI:** 10.1186/s10020-022-00534-0

**Published:** 2022-09-04

**Authors:** Forum Kayastha, Noah B. Herrington, Bandish Kapadia, Anirban Roychowdhury, Nahid Nanaji, Glen E. Kellogg, Ronald B. Gartenhaus

**Affiliations:** 1 McGuire Cancer Center, Hunter Holmes McGuire VA Medical Center, Richmond, VA USA; 2 Division of Hematology, Oncology, and Palliative care, Department of Internal Medicine, Massey Cancer Center, Virginia Commonwealth University School of Medicine, Richmond, VA USA; 3 Department of Medicinal Chemistry, Institute for Structural Biology, Drug Discovery and Development, Virginia Commonwealth University School of Pharmacy, Richmond, VA USA; 4 Department of Veteran Affairs, Maryland Healthcare System, Baltimore, MD USA

**Keywords:** DLBCL, eIF4A, Anti-tumor, Molecular modeling, Translational apparatus

## Abstract

**Background:**

Deregulated translation initiation is implicated extensively in cancer initiation and progression. It is actively pursued as a viable target that circumvents the dependency on oncogenic signaling, a significant factor in current strategies. Eukaryotic translation initiation factor (eIF) 4A plays an essential role in translation initiation by unwinding the secondary structure of messenger RNA (mRNA) upstream of the start codon, enabling active ribosomal recruitment on the downstream genes. Several natural product molecules with similar scaffolds, such as Rocaglamide A (RocA), targeting eIF4A have been reported in the last decade. However, their clinical utilization is still elusive due to several pharmacological limitations. In this study we identified new eIF4A1 inhibitors and their possible mechanisms.

**Methods:**

In this report, we conducted a pharmacophore-based virtual screen of RocA complexed with eIF4A and a polypurine RNA strand for novel eIF4A inhibitors from commercially available compounds in the MolPort Database. We performed target-based screening and optimization of active pharmacophores. We assessed the effects of novel compounds on biochemical and cell-based assays for efficacy and mechanistic evaluation.

**Results:**

We validated three new potent eIF4A inhibitors, RBF197, RBF 203, and RBF 208, which decreased diffuse large B-cell lymphoma (DLBCL) cell viability. Biochemical and cellular studies, molecular docking, and functional assays revealed that thosenovel compounds clamp eIF4A into mRNA in an ATP-independent manner. Moreover, we found that RBF197 and RBF208 significantly depressed eIF4A-dependent oncogene expression as well as the colony formation capacity of DLBCL. Interestingly, exposure of these compounds to non-malignant cells had only minimal impact on their growth and viability.

**Conclusions:**

Identified compounds suggest a new strategy for designing novel eIF4A inhibitors.

**Supplementary Information:**

The online version contains supplementary material available at 10.1186/s10020-022-00534-0.

## Background

Oncogenic signaling appears to dominate translational output at virtually every stage of cancer propagation for very specific and distinct cellular phenotypes (Sanchez-Vega et al. 2018; Hagner et al. 2010). With technological advancements, there is a growing recognition of selectivity in translational regulation mediated by core components of the mRNA biosynthetic apparatus (Truitt and Ruggero 2016). The regulation of messenger RNA (mRNA) translation in eukaryotic cells is critical for gene expression. It occurs principally at the initiation phase, primarily regulated by eukaryotic initiation factors (eIFs) (Pelletier et al. 2015). eIFs are fundamental for mRNA translation and act as the primary targets of numerous oncogenic signaling pathways to modulate gene expression. Thus, anti-tumor agents that strategically target the core components of protein synthesis and related signaling pathways represent novel therapeutic approaches with the potential to overcome resistance due to intra-tumor heterogeneity.

The most tightly regulated step of protein biosynthesis is the initiation of cap-dependent translation in which initiation factors bind to the 5-prime (5′) 7-methylguanosine (m7G) cap of mature mRNA to launch the translation of open reading frames (Mitchell et al. 2010). Cap-dependent translation is driven by the canonical heterotrimeric eIF4F complex, which catalyzes ribosome recruitment to mRNA and is comprised of eIF4G (scaffold protein), eIF4E (cap-binding protein), and eIF4A (ATP-dependent RNA helicase) (Lindqvist et al. 2008). eIF4A1 unwinds the secondary structure of RNA within the 5′ untranslated region (5′-UTR) of mRNA, a critical step necessary for the recruitment of the 43S preinitiation complex, and thus plays a vital role in initiating access to protein biosynthesis for the ribosomes (Raza et al. 2015). There are two mammalian isoforms of eIF4A involved in translation: eIF4A1 and eIF4A2 (Xue et al. 2021). The expression levels of eIF4A1 and eIF4A2 vary in a tissue-dependent manner. eIF4A1 is expressed more in proliferating cells compared to eIF4A2, which is dominantly expressed in growth-arrested differentiated cells, suggesting differential regulation of cell fate (Naineni et al. 2020). This observation that eIF4A1 and eIF4A2 have distinct biological functions in translational regulation in different subsets of cells and clinical conditions has supported the view that eIF4A1 is a rational cancer target.

The plethora of biochemical data on eIF4A1 (now referred to as eIF4A) reported in the last three decades has deciphered the detailed molecular mechanism of duplex destabilization by eIF4A and the governing principles of its minimal RNA helicase activity (Andreou and Klostermeier 2013). Genome-wide studies of the eIF4A-mediated translatome revealed that helicase regulates the expression of mRNAs encoding vital proteins associated with cell proliferation, cell survival, cell cycle progression and angiogenesis (Rubio et al. 2014; Wolfe et al. 2014). Critically, several reports emphasize that high expression levels of eIF4A significantly stimulate a cancer cell malignant phenotype (proliferation, invasion, migration and epithelial mesenchymal transition) and inhibit apoptosis (Modelska et al. 2015; Li et al. 2017; Liang et al. 2014; Gao et al. 2020). Thus, the effect of eIF4A up-regulation upon transformed cells appears to act via specific messages, perhaps in addition to a global up-regulation of translation, making eIF4A an attractive target for therapeutic intervention. In addition to the expected findings that eIF4A-dependent mRNAs contained longer 5′-UTRs with a greater degree of secondary structure, both Modelska et al. and Wolfe et al. observed that 5′-UTRs of eIF4A-dependent mRNAs are enriched with G-quadruplex motifs forming potential (Wolfe et al. 2014; Modelska et al. 2015).

Several natural compounds have been characterized that inhibit cap-dependent translation by specifically inhibiting eIF4A activity. These compounds include hippuristanol (Cencic and Pelletier 2016), pateamine A (PatA), and silvestrol (a rocaglate or “flavagline”) (Naineni et al. 2020). Rocaglate analogs are the most studied eIF4A inhibitors in the field (Chu et al. 2019). eFT226 (zotatifin), a structure-guided rocaglamide-inspired inhibitor, has entered Phase I clinical trials for solid tumors (Ernst et al. 2020). Recently, structural elucidation of a rocaglate [RocA]:eIF4A1:polypurine RNA complex revealed that rocaglates operate as interfacial inhibitors and make indispensable interactions with eIF4A1 and two adjacent RNA purine bases (Chu et al. 2019). However, all the compounds appear to act non-specifically upon eIF4A1 and eIF4A2. In this study, we report the discovery of three novel eIF4A1 inhibitors, RBF197, RBF203, and RBF208, which potentially bind to the same pockets as RocA but are chemically different. Although these compounds display a lesser degree of potency in their anti-tumor activity than RocA analogs, the observed effective dosage range of the compounds appears to be non-toxic to the transformed cells, and these molecules have scope for further medicinal chemistry design and development.

## Methods

### Molecular modeling studies

Prior to virtual screening, the crystal structure of the eIF4A1 complexed with a polypurine mRNA and RocA (PDB ID: 5ZC9) was obtained from the RCSB Protein Data Bank (Berman et al. 2000). RocA pharmacophore-based virtual screening of the MolPort and ZINC15 databases was conducted using the Unity module of the Sybyl-X 2.1.1 suite using its ‘Flex Search’ option. All docking studies were conducted in Genetic Optimisation for Ligand Docking (GOLD) (Jones et al. 1997) using the built-in ChemPLP scoring function. The high-throughput docking of our virtual screening hits was performed with aromatic ring center constraints and hydrogen bond donor and acceptor constraints, according to their positions in the original virtual screening pharmacophore. Higher-resolution dockings allowed at least 50 solutions per ligand while maintaining all but the original hydrogen bond donor constraints. Post-docking steepest descent energy minimizations were conducted using the Tripos force field in Sybyl-X 2.1.1 with a gradient of 0.02 kcal/mol, 100,000 iterations, Gasteiger-Hückel charges, and a dielectric constant of 8.0. Secondary scoring of eIF4A1-ligand complexes was conducted using the Hydropathic INTeractions (HINT) force field (Kellogg et al. 1991; Eugene Kellogg and Abraham 2000), a tool developed in our laboratory.

### Cell culture and transfections

Cells were procured from ATCC (DS, RC, Toledo, Farage, SUDHL-2, SUDHL-4) and DSMZ (OCI-Ly3)_._ GMO B-cells (lymphoblastoid cells) were purchased from the National Institutes of General Medical Sciences Human Genetic Mutant Cell Repository (Coriell Institute for Medical Research, Camden, NJ, USA). All DLBCL and GMO cells were grown in Roswell Park Memorial Institute (RPMI)-1640 except OCI-Ly3, grown in Iscove's Modified Dulbecco’s Medium (IMDM) with 10% FBS (Corning, Fetal Bovine Serum), and were maintained at 37 °C with 5% CO_2_. Hek293T/17 was cultured in Dulbecco’s Modified Eagle Medium (DMEM) with 10% FBS. Stable cells (293 T) were generated by PEI-mediated transfection, selected, and maintained with puromycin (1 mg/mL). Post selection, cells were cultured in DMEM containing 10% FBS. Cells were regularly passaged according to prescribed guidelines. Exponentially growing cells were treated with selected inhibitors and maintained at 37 °C, harvested at indicated time points for further analysis. The quality and authenticity of cell lines were performed regularly using regular mycoplasma testing and short tandem repeat (STR) profiling through the Nucleic Acid Research Facilities (NARF) at Virginia Commonwealth University (VCU), compared against known STR profiles.

### Reagents

RBF series small molecules were procured from MolPort, Inc platform, silvestrol: Medchem express, WST1: Dojindo Molecular Technologies Inc, phenazine ethosulfate, DMSO: Sigma-Aldrich, D-Luciferin, potassium Salt: Gold Biotechnology. All the other chemicals were procured from Fisher Scientific.

### Luciferase assays

Four tandem repeats of the (CGG)4 12-mer motif (GQs) or random sequence matched for length and GC content (random) were cloned into the pLenti-5′UTR-Luciferase (Wolfe et al. 2014). Empty firefly luciferase plasmid pLenti-5′UTR-Luc or blank with random sequence were used as controls (Wolfe et al. 2014). Briefly, after indicated treatments, cells were lysed in Triton Lysis Buffer (Baker and Boyce 2014). The cell lysate was mixed with luciferase assay reaction buffer (Kapadia et al. 2013), and luminescence was measured on a luminometer (Synergy H.T.X., Multi-Mode Reader).

### Cell proliferation and viability by WST − 1

Cell titrations were performed for optimal cell numbers. Five thousand cells for activated B-cell (ABC) DLBCL and 10,000 cells for germinal center B-cell (GCB) DLBCL were seeded in 96 well formats in 90 µL media. Cells were incubated for 24 h at 37 °C and 5% CO_2_. Compounds were dissolved in an appropriate solvent. Dilutions of compound (0.1, 0.3, 1, 3, 10, 30, 100 and 300 µM) were prepared containing stock concentration of 1% DMSO (10X). 10 µL of stock compound was added to each well in triplicates for each group and incubated for 72 h at 37 °C and 5% CO_2_ for the proliferation assay (Final concentration of the compounds: 0.01, 0.03, 0.1, 0.3, 1, 3, 10, 30 µM). 10 µL/well cell proliferation reagent (WST-1 (2 mg/mL), phenazine ethosulfate solution (0.21 mg/mL) dissolved in 1X PBS) was added and incubated for 1.5 h at 37 °C and 5% CO_2_ (Koyanagi et al. 2016). Absorbance was measured at 450 nm using a microplate (Synergy H.T.X., Multi-Mode Reader) reader.

### Assessment of viable cells

Exponentially growing cells were seeded in equal density (1 million). Post 12 h of seeding, cells were collected to the indicated time points and stained with trypan blue. The number of non-viable and viable cells was counted depending upon the intake of trypan blue in the hemocytometer using Thermo Fisher Countess III Automated Cell Counter (Kapadia et al. 2018).

### Phosphate release assay

A colorimetric assay to measure the phosphate released during ATP hydrolysis based on Malachite green was used to measure the activity of human eIF4A1 (1913204, ABM). The reaction buffers contained 50 mM potassium acetate, 20 mM MES pH6.0, 2 mM dithiothreitol (DTT), 0.1 mg/mL BSA and 100 ng/mL of whole yeast RNA (Type XI-C, Sigma-Aldrich) (Abdelkrim et al. 2018). The compounds were incubated at various concentrations with 20 ng human eIF4A1 in a reaction buffer for 30 min. Reactions were started by adding 50 µM ATP and then incubated at 37 °C for 2 h. Reactions were stopped by adding the solutions ∼ 60 mM in ethylenediaminetetraacetic acid (EDTA). The absorption at 630 nm was converted to phosphate concentration by a reference curve generated from a dilution series of a known phosphate concentration (1 mM Pi standard; SensoLyte^®^ MG Phosphate Assay Kit Colorimetric). Reaction velocities were determined by a linear regression fit of the phosphate generated against time. Only the initial linear phases of the curves were used. Velocities were determined for at least three independent experiments for each reaction condition.

### RNA unwinding assay

Custom-made RNA oligonucleotides were procured from Integrated DNA Technologies (IDT). RNA unwinding assay was undertaken with minor modifications as previously described (Andreou et al. 2017). Duplex RNA (5 µM) for unwinding reactions was prepared by annealing a 32mer RNA modified with cyanine 5 (Cy5) at its 5′-end (5′Cy5- CGAGG UCCCA AGGGU UGGGC UGUUC GCCCA UU-3′) and a complementary 9mer modified with a cyanine 3 (Cy3) at the 3′-end (5′-UUGGGACCU-Cy3–3′) in 25 mM Hepes/KOH, pH 7.4. The mixture was heated to 96 °C for 2 min, slowly cooled to room temperature, and incubated on ice for 15 min. A 9-nucleotide loop connecting the duplex strands was introduced for the single turnover condition. Experiments were performed in 30 mM HEPES/KOH pH 7.4, 100 mM KOAc, 3 mM Mg(OAc)_2_, 2 mM DTT at 25 °C with 100 nM duplex RNA, a tenfold molar excess of unlabeled 9mer RNA to trap the released 9mer (5′-AGG UCC CAA-3′), and 5 µM of human eIF4A1. The compounds were incubated with human eIF4A1 for 30 min at 25 °C. Unwinding reactions were initiated by adding 1 mM ATP after obtaining a stable fluorescence signal. Kinetic readings were captured for 30 min (The stability of the signal was noted at 3 min, however, to ensure that the reaction is complete (all molecules bind with eIF4A RNA complex), we took the reading till 30 min. Post 30 min, another duplex (100 nM) was added to the mixture, and an increase in the fluorescence was measured after 3 min. Second readings (3 min after the second duplex was added) were normalized with the first reading (30 min). Here the assumption is that the compound will clamp eIF4A: RNA complex and hampers the recycling of enzymes for its helicase activity. Thus, the enzyme that is freely available for the activity will act on the newly added substrate. Excess molar ATP was used (50 µM for phosphatase assay compared to 1 mM in this assay). All measurements were conducted in an Infinite® 200 PRO (Tecan). Cy3 fluorescence was excited at 554 nm (1 nm bandwidth), and Cy5 fluorescence was detected at 666 nm (3 nm bandwidth) in 10 s intervals with an integration time of 0.1 s. All measurements were repeated at least three times, and the increase in the fluorescence was calculated in the presence or absence of the inhibitors.

### Surface sensing of translation (SUnSET) assay

SUnSET assay was performed as per the manufacturer's recommendations (Kerafast) as previously reported (Kapadia et al. 2018). In brief, cells were pulse-labeled with puromycin (1 µg/mL) for 30 min. Post-treatment, cells were washed with ice-cold PBS, lysed, and probed (10 µg of protein) with an anti-puromycin antibody. Signals were normalized by indicated loading control in each set.

### Immunoblotting

Cells were lysed in RIPA buffer (50 mM TRIS pH 7.5, 150 mM NaCl, 0.1% SDS, 0.5% sodium deoxycholate, 1% triton X-100, 1 mM EDTA, and 1 mM EGTA, 1 mM sodium orthovanadate, 1 mM sodium fluoride, 1 × protease inhibitor (Sigma-Aldrich), phosphatase inhibitor cocktails #2 and #3 (Sigma-Aldrich), and 1 mM PMSF) (Kapadia et al. 2018). Cells lysate were quantified using Bradford reagents and equal amount of protein was separated on a BOLT 4–12% Bis–Tris gradient gel (Life Technologies) and probed with the following antibodies: eIF4A (sc-377315 or sc-14211, 1:1000), eIF4E (SCBT, sc-9976, 1:1000), cMyc (SCBT, sc-373712 or SC-764, 1:1000), Bcl2 (CST # 2870 or SCBT, sc-509, 1:1000), Bcl6 (CST, #5650, 1:1000), Parp-1 (SCBT, sc-7150, 1:1000), Actin (SCBT, sc-1615, 1:1000), Gapdh (Abcam, ab8245, 1:10,000), Vinculin (Sigma, V9131, 1:10,000), Cdk7 (sc-529, 1:1000) Nrf2 (CST, #12721, 1:1000) and Card11 (CST, #4435, 1:1000). Densitometry analyses were performed using Image Studio (Licor Biosciences) and presented as ratio of target band signal intensity to Gapdh/Actin/Vinculin band signal intensity.

### Clonogenic methylcellulose assays

Lymphoblastoid cells (GMO 17220B, 1528, 13604), DLBCL cells (ABC-DLBCL: OCI-Ly3, DS, and GCB-DLBCL: Farage, RC, Toledo) cell lines were utilized for colony formation assay. Briefly, cells (25–50 × 10^3^) were mixed methylcellulose (RnD) conditioned IMDM with 20% FBS (Kapadia et al. 2018). Cells were then treated with the respective compounds or DMSO, and colonies were visualized and counted at 15-day post treatment.

### Immunohistochemistry

To study the expression pattern of the eIF4A1 protein, immunohistochemical staining was performed using Biocare Medical Intellipath F.L.X. auto-stainer. Briefly, tissue microarray (TMA) slides (US Biomax, LY1001B, LY1001C E069, LM801 280, Y10001D SD43 and LY800B B040) were baked at 65 °C and deparaffinized (X1-10 min, X2-10 min) by using Xylene (Fisher Scientific) and subsequently hydrated by sequential incubation in ethanol (100% EtOH-5 min, 70% EtOH-5 min, 50% EtOH-5 min, H_2_O-5 min). Antigen retrieval was performed using a Decloaking Chamber™ NxGen (Biocare Medical) with pre-heated 1X Borg Declokar buffer (Biocare Medical, USA) at 95 ºC for 30 min. Slides were then loaded into the Intellipath FLX™ machine, and blocking of the endogenous peroxidase was performed by incubation with intelliPATH FLX™ Peroxidase Blocking Reagent (Biocare Medical) for 10 min. Later tissue sections were blocked with intelliPATH™ Background Punisher (Biocare Medical) for 5 min and incubated with the primary antibodies against anti-eIF4A1 (1:200) (rabbit monoclonal ab31217; Abcam, Cambridge, MA) for 1 h (Zhao et al. 2021). The sections were washed with TBS Automation Wash Buffer (Biocare Medical) and incubated with MACH 2 Universal HRP-Polymer polymer (Biocare Medical) for 30 min. Immunoreactivity was visualized by intelliPATH FLX™ DAB Chromogen Kit (Biocare Medical) followed by intelliPATH™ Hematoxylin (Biocare Medical) counterstain and mounted with EcoMount (Biocare Medical). Based on the staining intensity, samples were scaled between 1 and 4, as discussed earlier (1 means low staining intensity while 4 represents the highest staining intensity) (Kapadia et al. 2022).

### Analysis of eIF4A1 expression in publicly available DLBCL datasets

UACLAN (http://ualcan.path.uab.edu/) is an extensive resource to evaluate tumor data, primarily The Cancer Genome Atlas (TCGA). The expression of eIF4A1 in TCGA DLBCL samples (n = 41) was extracted using the UACLAN database (Chandrashekar et al. 2017; Chandrashekar et al. 2022). We obtained eIF4A1 in naive B cells from healthy individuals (n = 91) from the DICE [Database of Immune Cell Expression, Expression quantitative trait loci (eQTLs), and Epigenomics] database, which is a comprehensive resource of expression and epigenomic profiles of different types of human immune cells (Schmiedel et al. 2018). We also mined the expression of eIF4A1 in other publicly-available DLBCL datasets (Schmitz et al. 2018), GSE10846 (Lenz et al. 2008; Cardesa-Salzmann et al. 2011) and GSE87371 (Dubois et al. 2017; Dubois et al. 2019). Finally, prognostic implications of eIF4A1 in DLBCL were assessed using the Genomic Data Commons (GDC) dataset (Schmitz et al. 2018).

### Quantitative reverse transcription real-time polymerase chain reaction

RNA was extracted from DLBCL cells using TRIzol (Invitrogen Corporation). cDNA was reverse transcribed from 1000 ng RNA using the High-Capacity RNA-to-cDNA™ Kit (Thermo Scientific). All primer sequences included in the study are listed in Additional file 2: Table 1. Quantitative reverse transcription real-time polymerase chain reaction (qRT-PCR) methods were developed on a Power SYBR Green Master Mix (Thermo Scientific) according to the manufacturer’s instructions. The mRNA expressions were analyzed using the 2 − ΔCT method (Kain et al. 2015).

**Table 1:** Key interactions in the eIF4A1—RocA structure

Residue/nucleotide	Residue/nucleotide atom	RocA atom	Interaction score	Interaction type
GLN195	NE2	O1	240	Hydrogen bond
G8	N7	O6	87	Hydrogen bond

### Statistics

Data were analyzed using GraphPad Prism 9. Values were expressed as mean ± S.D. of a minimum of three independent experiments. Wilcoxon signed-rank test was used to compare the data sets between naïve GCB B-cells and DLBCL samples; p < 0.05 was considered significant. The unpaired Student’s *t*-test was used to compare the two groups. One-way ANOVA followed by either Dunnett’s or Bonferroni’s post hoc analysis compared more than two groups; p < 0.05 was considered significant. Hill coefficient was calculated for the concentration–response curves.

## Results

### Expression of eIF4A1 predicts poor outcome in diffuse large B cell lymphoma

**Fig. 1:**
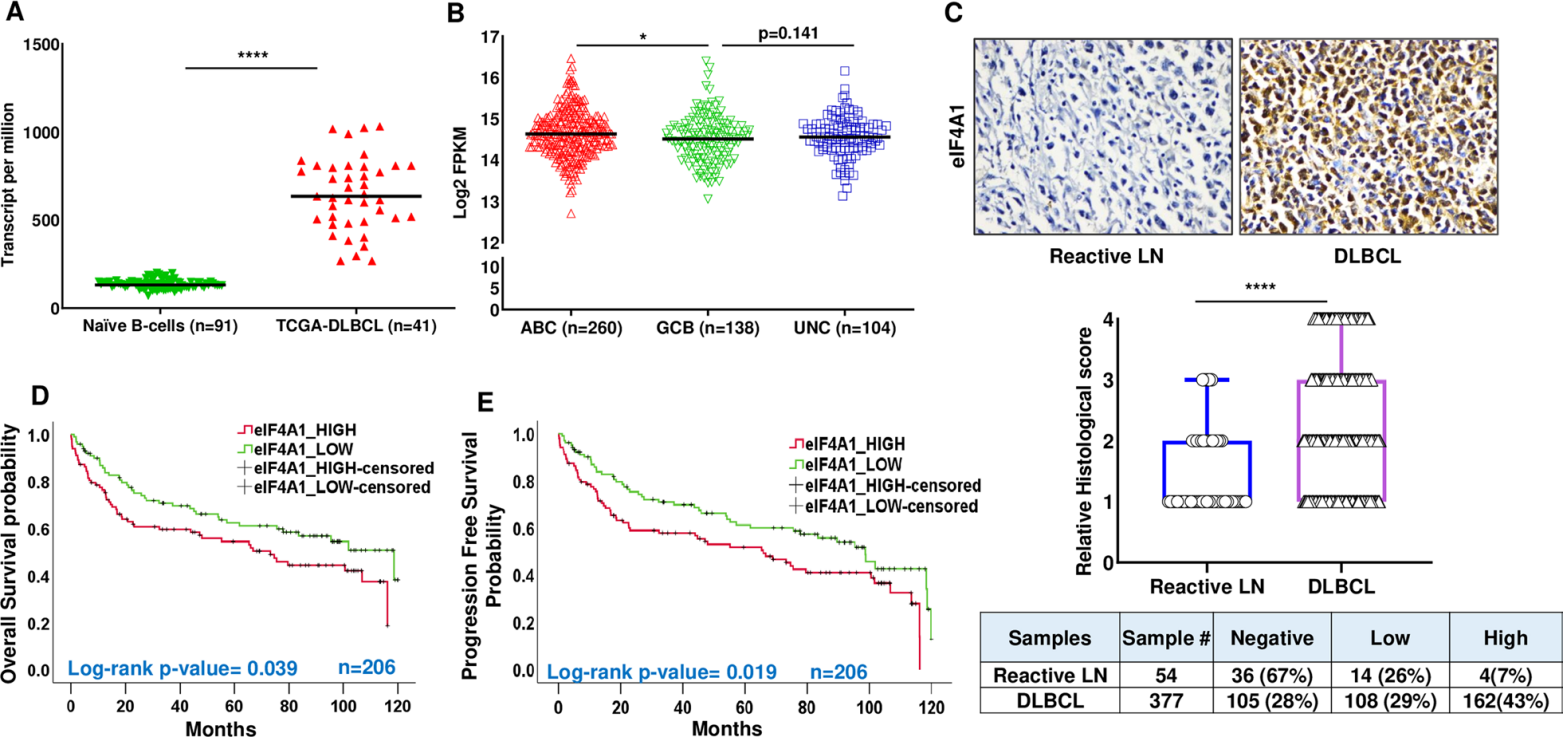
Clinicopathologic evaluation of eIF4A1. **A** Representative plots show RNA-seq expression profiles of eIF4A1 in naïve B-cells (n = 91) (obtained from DICE database https://dice-database.org/) compared with DLBCL (n = 41) in TCGA dataset. eIF4A1 showed significantly higher expression in tumor samples compared with control. The Y-axis represents transcript per million (TPM) values. ****p < 0.0001. **B** Comparison of RNA-seq data of eIF4A1 in molecular subgroups using a publicly available large dataset of patients with DLBCL. eIF4A1 showed significantly higher expression in ABC-DLBCL (n = 260) subgroups compared with GCB-DLBCL (n = 138) and UN-DLBCL (n = 104), *p < 0.05. The values are represented in log base 2 of fragments per kilobase of exon per million mapped fragments (FPKM). **C** Representative immunohistochemistry image of commercially procured (US Biomax., Inc) TMA slides stained with eIF4A1 antibody. Representative scatter plots showing the stained signals of eIF4A1 in reactive LN compared to DLBCL samples. Statistical analysis was performed using Wilcoxon signed-rank test (unpaired two-tailed), ****p < 0.001 vs. reactive LN. Summary chart for DLBCL and normal LN. − ve: no staining detected, low: 1–2 staining density, high: 3–4 staining density. **D** eIF4A1 expression was found to be significantly (*p* = 0.039) associated with OS of patients with DLBCL in the publicly available dataset (n = 206). Patients with a lower median expression of eIF4A1 showed a better prognosis than patients having higher median expression. **E** eIF4A1 expression was also found to be significantly (*p* = 0.019) associated with the PFS in the same cohort of patients with DLBCL having a similar observation

To elucidate the pathophysiological relevance of eIF4A1 in DLBCL, we examined the publicly available datasets. Analyzing the expression profile of eIF4A1 in the Database of Immune Cell Expression (DICE), Expression quantitative trait loci (eQTLs), Epigenomics (Schmiedel et al. 2018), and The Cancer Genome Atlas (TCGA) (Chandrashekar et al. 2017) datasets, we observed a robust increase (*p* < 0.0001) in the transcript levels of eIF4A1 in DLBCL samples compared to naïve B-cells (Fig. 1A), supporting the relevance of eIF4A1 in lymphomagenesis. Given the substantial variability and unique heterogeneity/biology within DLBCL, this lymphoma subgroup is further classified as Activated B-cell (ABC), Germinal Center B-cell (GCB), and Unclassified (UNC) DLBCL based on its expression profile (Menon et al. 2012). In support of our observation, ABC-DLBCL cohorts (n = 260) [which have a worse outcome with standard immune-chemotherapy compared to GCB-DLBCL (Nowakowski and Czuczman 2015)] display higher expression of eIF4A1 compared to GCB-DLBCL (n = 138) (*p* = 0.032) or UNC-DLBCL (n = 104) (*p* = 0.191) (Fig. 1B). In agreement with these data, transcriptomic profiles [GSE10846 (Lenz et al. 2008) and GSE87371 (Dubois et al. 2019)] showed that eIF4A1 mRNA was expressed at a higher level in ABC-DLBCL (n = 250) compared with GCB-DLBCL (n = 268) or UNC-DLBCL (n = 64) (Additional file 1: Fig. S1). To further validate this observation, we stained primary DLBCL specimens from commercially procured DLBCL tissue microarrays (US Biomax., Inc). In coherence with the above data, the protein levels of eIF4A1 were robustly detected in DLBCL samples (n = 377) compared to Reactive Lymph Nodes (LN) (n = 54) (Fig. 1C). Staining DLBCL samples displayed 72% expression of eIF4A1 while that of reactive lymph node was 33%. Collectively, eIF4A appears to be upregulated in DLBCL.

To further investigate the clinical importance of eIF4A1 in lymphoma progression, the prognostic value of eIF4A1 gene expression was determined using publicly available datasets (Chandrashekar et al. 2017; Chandrashekar et al. 2022; Schmiedel et al. 2018), employing a cox p-value < 0.05. As shown in Fig. 1D, E, eIF4A1 was significantly associated with overall survival (OS) and progression-free survival (PFS) of patients with DLBCL. Patients with a higher median expression of eIF4A1 showed shorter survival periods than those with lower median expression. (Fig. 1D, n = 206, *p* = 0.039). Consistently, patients with higher expression of eIF4A1 have a shorter progression-free interval than patients with low expression of eIF4A1 (Fig. 1E, n = 206 *p* = 0.019). Altogether, the above clinical data endorses the claim that higher eIF4A1 gene expression is associated with poor survival and more aggressive clinicopathological features, supporting our notion that eIF4A1 is a promising therapeutic target in DLBCL.

### Structure-based drug screen identifies new inhibitors of eIF4A1

**Fig. 2:**
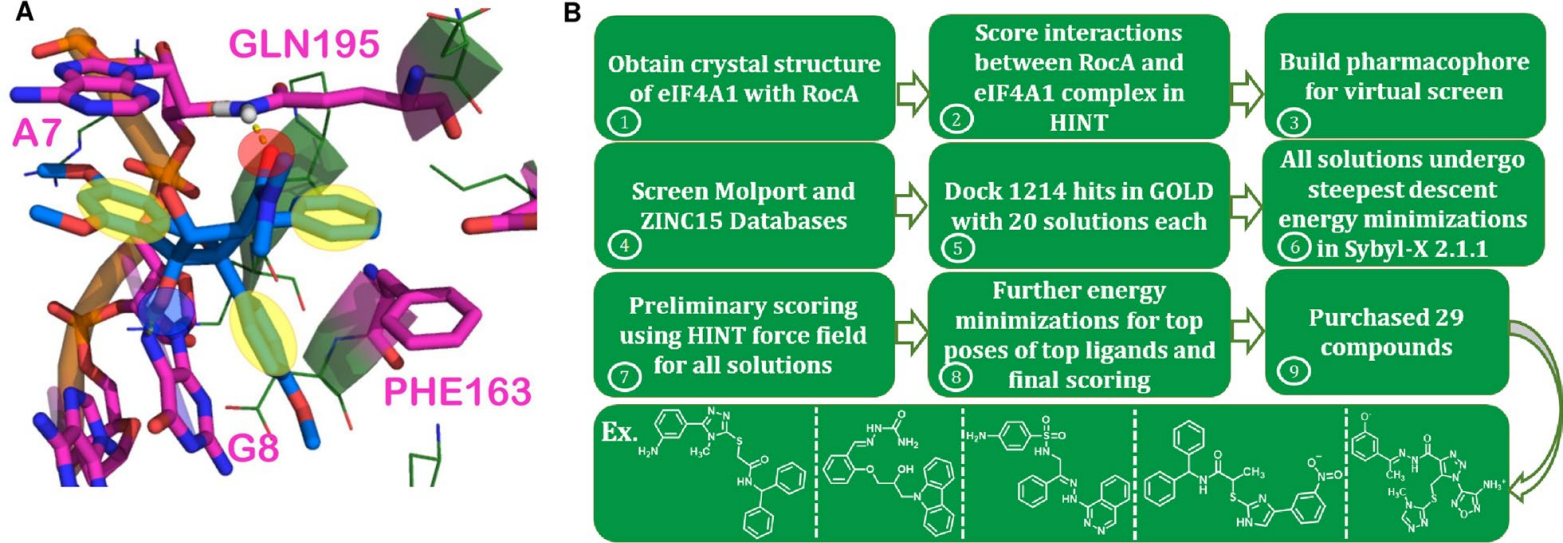
Flow chart representing the process taken to identify new small molecule inhibitors for eIF4A. **A** Model of RocA used to define important pharmacophore features used in pharmacophore-based virtual screening experiment. In yellow circles are shown regions defining positioning of aromatic rings. The red circle indicates a hydrogen bond acceptor feature, while the blue circle indicates a hydrogen bond donor feature **B** Workflow for virtual screening strategy that identified RBF98 as the top hit. Stages for this workflow included obtaining the crystal structure of eIF4A1 complexed with RocA, scoring interactions between these two species, constructing and implementing the virtual screening pharmacophore, high-throughput molecular docking, energy minimizations of solutions, preliminary scoring of solutions in HINT, and final energy minimizations and scoring, followed by the purchase of the 29 top-scoring hits

Our search for novel inhibitors of eIF4A1 began with a structure-based virtual screen of a potential binding site of eIF4A1. Using a crystal structure [PDB ID: 5ZC9 (Iwasaki et al. 2019)] of eIF4A1 complexed with a polypurine mRNA strand and RocA, which is a natural product inhibitor of eIF4A. To establish what features of RocA to use for the basis of our virtual screen, we utilized the HINT force field (Kellogg et al. 1991; Eugene Kellogg and Abraham 2000) to determine the molecular features of RocA most responsible for its binding. Briefly, HINT is a scoring function based on the free energy associated with solvent partitioning between 1-octanol and water. It has been used in numerous studies involving interactions between and amongst proteins, polynucleotides, and small molecules (Obaidullah et al. 2018; Spyrakis et al. 2013; Chen et al. 2008). In addition to π-π stacking interactions between the mRNA bases of the polypurine strand and with PHE163 in literature reported by Iwasaki et al. (Iwasaki et al. 2019), we noted two key hydrogen bonding interactions involving the ligand and GLN195, as well as G8 (Fig. 2A). Table 1 lists these crucial interactions that we concluded contributed most to RocA’s binding and activity.

The hydrogen bond between O6 of RocA and G8 of the mRNA is consistent with Iwasaki et al.’s structural studies. Thus, the aromatic rings of RocA, along with its acceptor O1 and donor O6, were used as pharmacophore features in a virtual screen for new inhibitors (Fig. 2B). Virtual screening was performed using the ‘Flex Search’ option of the Unity (Hurst 1994) suite in Sybyl-X 2.1.1. A total of 1218 hits from the screen underwent high-throughput docking in GOLD 5.6.1 with 20 solutions per ligand. All solutions were triaged into the HINT force field for secondary scoring. The top 29 scoring compounds (Additional file 1: Table S2) from HINT were purchased and further assayed for activity.

**Fig. 3:**
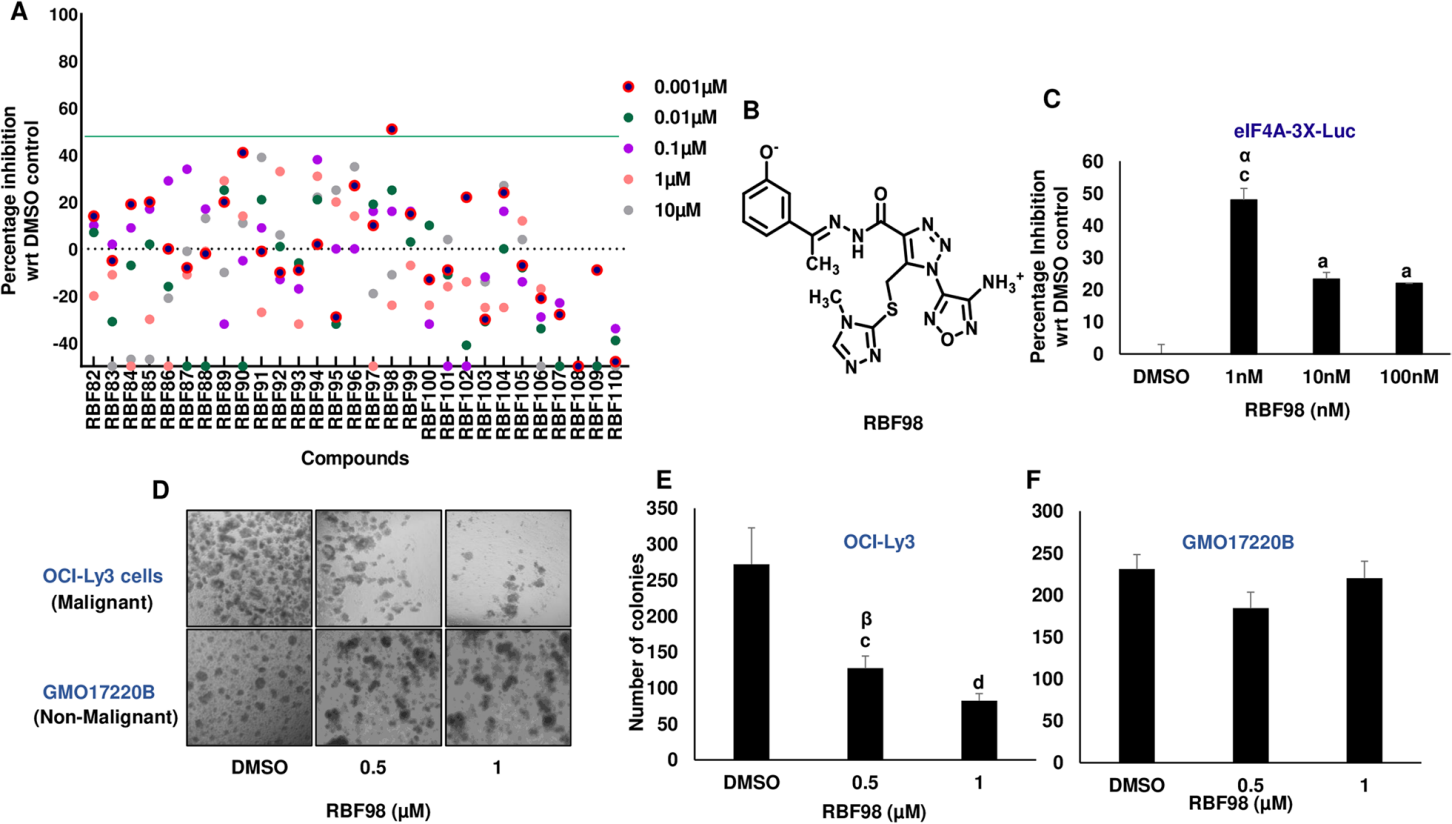
eIF4A specific high throughput screen identifies small molecules with inhibitory effect. **A** Scatterplot of primary screen results. A total of 29 compounds were tested and luciferase signal reduced by ≥ 50% compared to control were identified and considered active. Luciferase activity results are expressed relative to values obtained in the presence of vehicle controls. Percentage inhibition was calculated and plotted in a scatter plot, n = 3 biological replicates performed Mean ± SEM. **B** Structure of RBF98, a candidate inhibitor. **C** Percentage inhibition observed in the treatment of RBF98 at various concentrations in eIF4A-3X-Luciferase Hek293T/17. **D** Effect of RBF98 on DLBCL colony formation. Representative image of the colony formation in OCI-Ly3 (malignant) and GMO17220B (non-malignant) cells. The total number of colonies grown in **E** OCI-Ly3 and **F** GMO17220B cells upon treatment with 0.5 and 1 µM of RBF98. Statistical analysis was performed using one-way ANOVA followed by Bonferroni’s correction analysis. ^a^p < 0.05; ^c^p < 0.001, ^d^p < 0.0001 vs DMSO control groups, ^α^p < 0.05, ^β^p < 0.001, ^¥^p < 0.0001 vs 1 mM RBF98 treated groups

**Fig. 4:**
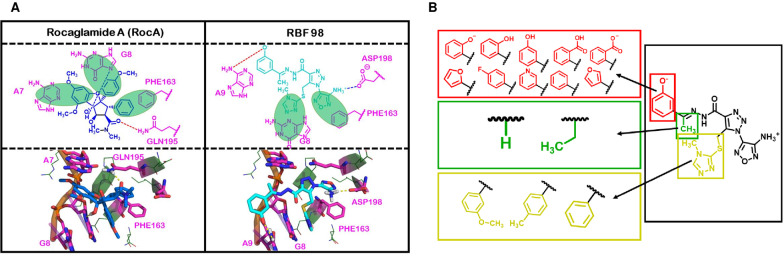
Interaction differences between RocA and docked RBF98. **A** Interaction environments for RocA and RBF98. The above two panels show stick representations of the interactions made between RocA and RBF98 and their surrounding environments. Green, transparent ovals are used to two-dimensionally represent possible π-π stacking interactions between the ligands and surrounding residues. Dashed lines between the ligands and surrounding residues are used to indicate hydrogen bonding, where the color indicates the donor/acceptor character of the ligand atom (blue = donor; red = acceptor). **B** Summary of results from secondary screening of RBF98 analogues, purchased from MolPort. Boxes surrounding different moieties of RBF98 correspond by color to the larger boxes containing functional groups that were sampled as part of this secondary screen in different combinations

To rapidly evaluate the inhibitory ability of the selected novel eIF4A inhibitors, we took advantage of an in-cell high throughput eIF4A-3X luciferase assay (Wolfe et al. 2014). The luciferase-based reporter assay with 5’UTR of eIF4A-sensitive four tandem repeats of the (CGG)_4_ 12-mer motif (GQs) driven by beta-actin promoter was used as a platform for primary screening in Hek293T/17 stables cell lines (Additional file 1: Fig. S2A). Cofactors like eIF4B stimulate the activity of eIF4A1. However, the eIF4A regulated luciferase readout was minimally dependent on eIF4B (Additional file 1: Fig. S2B). Similar experiments were performed with silvestrol as an internal control (Additional file 1: Fig. S2C), suggesting that the consensus sequence is highly reliant on eIF4A. The 29 commercially available hit compounds were used for the initial primary screen at concentrations ranging from 1 nM to 10 µM. Luciferase readout greater than 50% was considered the cut-off value for the screen. We observed that RBF98 (showed around 50% inhibition with respect to the DMSO control at 1 nM concentration (Fig. 3A–C). Interestingly, the percentage decrease in the luciferase readout was less than 10% in blank and empty luciferase groups, suggesting high specificity for the compound (Additional file 1: Fig. S3A) in limiting eIF4A-driven translation. It should be noted that the compound RBF98, at higher concentrations, showed a decrease in eIF4A inhibition, probably due to various physicochemical properties such as reduced solubility, etc. The ability of compound RBF98 to preferentially target eIF4A-sensitive luciferase with a readout similar to silvestrol provides promising evidence that RBF98 inhibits eIF4A and not another protein in the general translational apparatus and also binds in a manner similar to silvestrol. After analyzing its highest-scoring docked pose, it was concluded that RBF98 might adopt a similar binding mode to RocA, with three of its aromatic rings forming π-π stacking interactions with two adjacent nucleotide bases and PHE163, which seem to be crucial for rocaglate activity. Further, this docked pose of RBF98 shows that its phenoxide moiety may form a hydrogen bond with G8 of the RNA strand and a novel ionic interaction between its ammonium group and ASP198. This previously unobserved interaction may be integral for achieving improved drug-like properties over rocaglamate-based inhibitors (Fig. 4A). Additional biochemical testing was performed with RBF98 to investigate the direct inhibition of the compound on eIF4A’s helicase activity. Here, we ran an inorganic phosphate release assay to directly measure the eIF4A1 ATP-dependent RNA helicase activity using a stable mixture of yeast RNA. To achieve a maximal signal-to-noise ratio in this endpoint assay, we optimized the amount of enzyme and the incubation time (Additional file 1: Fig. S3B, C). RBF98 showed a dose-dependent decrease with an inhibitory effect at ~ 50% at a concentration of 0.3 µM compared with the DMSO control (Additional file 1: Fig. S3D).

We next analyzed RBF98 impact on a cellular proliferation assay. The compound reduced the cellular proliferation of DLBCL cells at 0.5 µM and 1 µM concentrations. Silvestrol was again used as a positive control (Additional file 1: Fig. S4A). Cellular proliferation at lower concentrations had minimal impact on DLBCLs (data not shown). For further insight into the effect of RBF98, we performed a colony formation assay in the OCI-Ly3, Toledo (malignant cell lines), and lymphoblastoid cells (GMO 17220B, 1528, 13604, non-malignant cell lines). We observed that RBF98 significantly decreases colony formation in malignant cell lines in a dose-dependent manner (Fig. 3D, E, Additional file 1: Fig. S4B). Notably, treatment of lymphoblastoid cells had minimal impact on their proliferative capacity indicating that the compound may have a potential non-toxic effect on non-malignant cells, addressing a major limiting factor of the currently available eIF4A inhibitors (Fig. 3D, F, Additional file 1: Fig. S4B). To further explore the molecular insights of RBF98’s activity, we pulse-labeled DLBCL cells with puromycin after treatment with the compound. Immunoblotting with anti-puromycin revealed a concentration-dependent decrease of puromycin labeling along with the protein levels of eIF4A, but minimal changes in eIF4E, indicating an overall reduction in the translation capacity of the cells (Additional file 1: Fig. S4C). Further, the expression of eIF4A-dependent genes, cMYC (Zhang et al. 2020) and CyclinD1 (Stoneley and Willis 2015), was reduced similarly (Additional file 1: Fig. S4C).

**Fig. 5:**
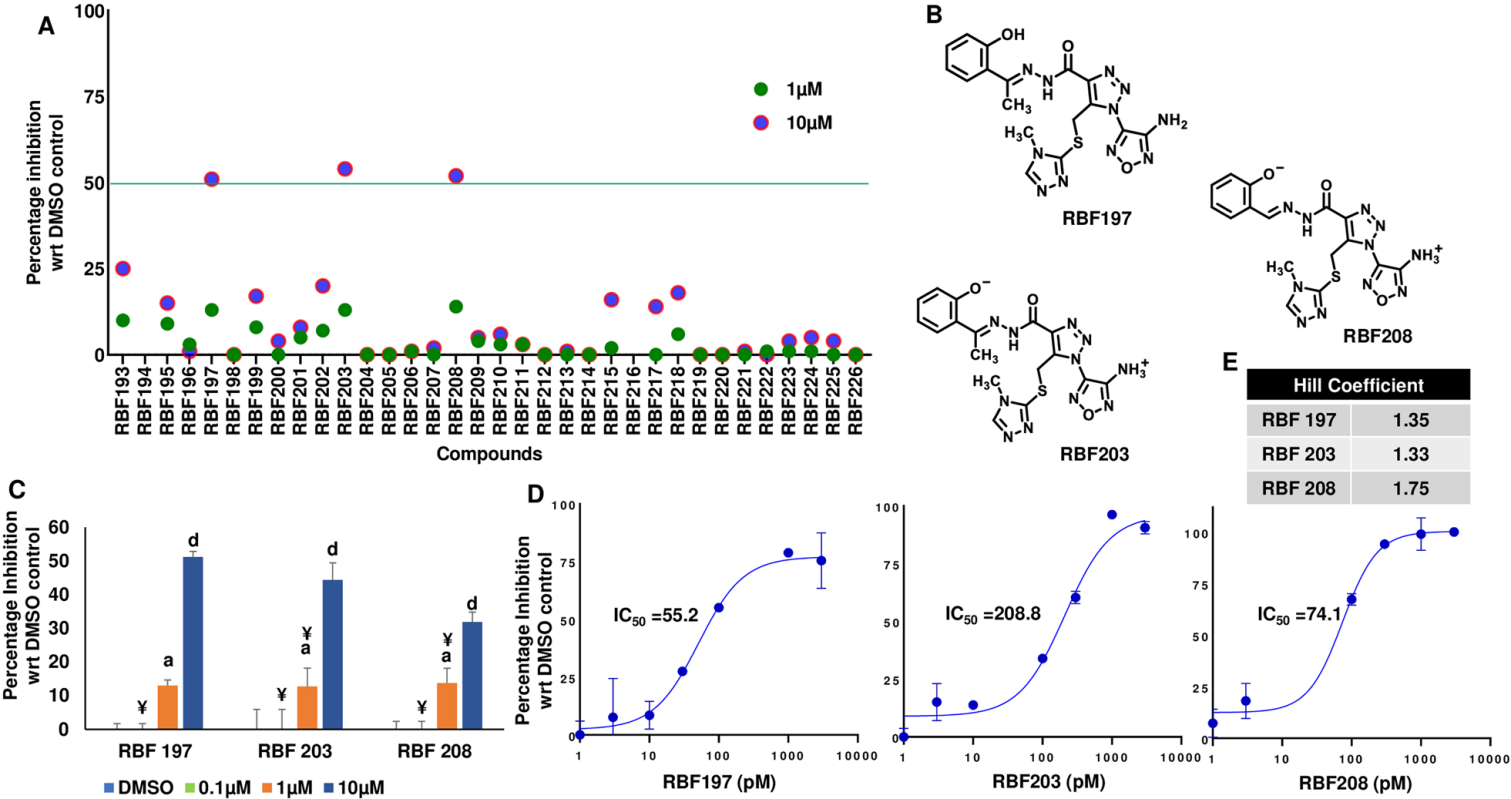
Secondary screen and evaluation of RBF98 analogs. **A** A total of 34 compounds that inhibited Luciferase signal by ≥ 50% compared to control were identified. Luciferase activity results are expressed relative to values obtained in the presence of vehicle controls. Percentage inhibition was calculated and plotted in a scatter plot, n = 3 biological replicates performed Mean ± SEM. **B** Structures of RBF197, RBF203, and RBF208, potent candidate inhibitors. **C** Representative plots of percentage inhibition values of luciferase activity on the treatment of RBF 197, RBF203, and RBF208 at 0.1, 1, and 10 µM in eIF4A1-3X-Luciferase in Hek293T/17 cell lines for 24 h (n = 3). Statistical analysis was performed using one-way ANOVA followed by Bonferroni’s correction analysis. ^a^p < 0.05; ^c^p < 0.001, ^d^p < 0.0001 vs DMSO control groups, ^α^p < 0.05, ^β^p < 0.001, ^¥^p < 0.0001 vs 10 μM treatment groups. **D** Concentration–response curves of percentage inhibition of human eIF4A1 in-vitro activity on the treatment of RBF197, RBF203, and RBF208, compared to DMSO control by measurement of inorganic phosphate released (SensoLyte Kit). IC_50_ values observed were 55.2, 208.8, and 74.1 pM, respectively. **E** Hill coefficient values for the concentration–response curves

After screening the initial set of 29 compounds, we pursued analogs of RBF98 in the hope of identifying purchasable compounds with better or comparable activity to it, our most potent hit. Using a similarity search function based on Tanimoto indices (Rogers and Tanimoto 1960) built into the MolPort website, we identified 34 analogs (Additional file 2: Table S3) that had chemical similarities to RBF98. Generally, the compounds from this round of screening structurally differed in three positions from RBF98, which allowed us to probe aspects of the structure–activity relationship of our hit (Fig. 4A). We mainly focused on altering these positions because they were the most accessible changes, based on the collection of compounds commercially available from MolPort. Figure 4B illustrates some of the different structural variations in these positions. After the luciferase readout, the primary screen was applied to the 34 compounds we obtained, RBF197, RBF203, and RBF208 (Fig. 5A, B), which displayed a dosage-dependent decrease in luciferase readout (Fig. 5C). More importantly, all three hit molecules do not show more than 15% inhibition of blank (Additional file 1: Fig. S5B) and empty luciferase readout (Additional file 1: Fig. S5A), implicating a specific inhibitory effect on eIF4A dependent activity. Most of the compounds available for purchase from our virtual screen and that we assayed showed alterations to the *m*-phenoxide moiety of RBF98, thus allowing us to probe this region extensively (Fig. 4B). The other two regions of the RBF98 first-round lead remain largely unexplored.

To further corroborate our findings, we subjected these three new hits to a kinetic assessment using an in-vitro inorganic phosphate assay. To our surprise, the selected three compounds potently inhibited eIF4A helicase activity in a dose-dependent manner with IC_50_ values in the picomolar range and with Hill coefficients 1.35, 1.33 and 1.75 indicating single-molecule binding without aggregating effects (Fig. 5D, E).

### Novel eIF4A inhibitor blocks cell proliferation and impedes overall translation in DLBCL

To determine if the most potent compounds exert inhibitory activity in DLBCLs, we first subjected a panel of DLBCL cells to a WST -1 cell viability assay. This assay quantitates the number of living and metabolically active cells by measuring the cleavage of tetrazolium salts by intracellular enzymes. As shown in Table 2, all the hit compounds decreased the cellular viability of DLBCL cells with a half maximal effective concentration (EC_50_) in the low micromolar range (Additional file 1: Fig. S6). To our surprise, SUDHL2, which harbors a mutation in A20, SOCS1, and TP53, was insensitive to the eIF4A inhibitors (Juskevicius et al. 2018). Additional analogs with varying potencies in the luciferase readout assays were tested in the cell viability assay to investigate if helicase inhibition tracked DLBCL WST1 inhibition, and to ensure that cell viability did not decrease due to the general toxicity of the inhibitor scaffold (Additional file 2: Table S4). As anticipated, these molecules do not dramatically affect the DLBCL cellular viability; thus, minor substitution to our selected compounds that hampers their eIF4A inhibitory capacity also displays a minor reduction in potency of DLBCL cellular viability.

**Table 2:** IC_50_ values of RBF197 and RBF208 in a panel of six DLBCL cell lines using WST-1 assay

DLBCL cell lines	RBF 197 (µM)	RBF208 (µM)
OCILy3	0.4	0.9
SUDHL4	3.2	4.5
Farage	2.4	2.9
DS	2.8	4.3
RC	1.9	5.2
SUDHL2	> 30	> 30

**Fig. 6:**
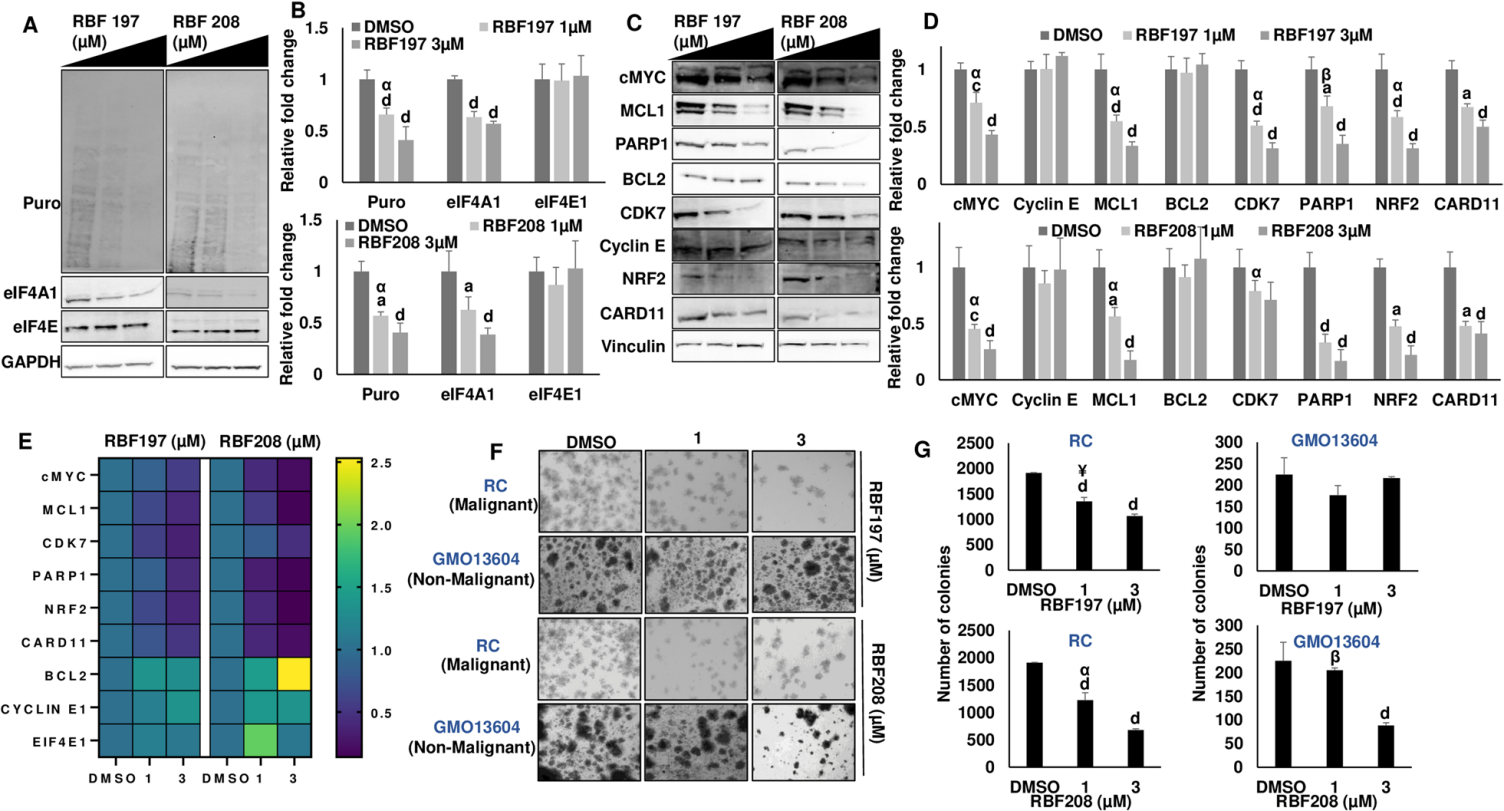
RBF197 and RBF208 decreases overall translation and eIF4A1 dependent pathway proteins in double-hit lymphoma. **A** SUnSET assay was performed by exposing cells to puromycin (1 μg/mL), post compound treatments for 30 min, and subsequently lysed. Representative immunoblots were probed with anti-puromycin, anti-eIF4A, and anti-eIF4E. GAPDH was probed as an internal loading control. **B** Relative fold change in expression levels with on treatment of RBF197 or RBF208 at 1 and 3 µM concentrations. **C** Representative immunoblots of cMYC, MCL1, PARP1, BCL2, CDK7, NRF2, Cyclin E and CARD11 on treatment with RBF197 and RBF208 at 1 and 3 µM concentrations respectively. Vinculin was used as a loading control. **D** Relative fold change in protein levels of the above-mentioned proteins with on treatment of RBF197 and RBF208, respectively. **E** Heat map of translation efficiency values for RBF197 and RBF208 in RC cell line. **F** Effect of RBF197 and RBF208 on DLBCL colony formation. Representative image of the colony formation in RC (malignant) and GMO13604 (non-malignant) cells. **G** The total number of colonies grown in RC, and GMO13604 cells upon treatment with 1 and 3 µM of RBF197 and RBF 208, respectively. All treated groups were normalized with DMSO controls and expressed as mean ± SD (n = 3). Statistical analysis was performed using one-way ANOVA followed by Bonferroni’s correction analysis. ^a^p < 0.05; ^c^p < 0.001, ^d^p < 0.0001 vs DMSO control groups, ^α^p < 0.05, ^β^p < 0.001, ^¥^p < 0.0001 vs 3 μM treatment groups

Next, we tested two compounds, RBF197 and RBF208, for their detailed mechanistic profiling on eIF4A-dependent transcripts in DLBCL. A cellular translation assay was performed to confirm the ability of these molecules to impede cellular protein biosynthetic machinery and to correlate the cellular viability by small molecules to eIF4A inhibition. We treated DLBCLs RC (GCB, Double-Hit) and OCI-LY3 (ABC) cell lines with the compounds for 16 h, followed by a pulse labeling for 30 min with puromycin. Inhibiting eIF4A activity in DLBCL cells showed a significant dose-dependent decrease in overall protein translation output (Fig. 6A, B, Additional file 1: Fig. S7A, 7B). The protein levels of eIF4A, but not eIF4E1, showed a dosage-induced decrease (Fig. 6A, B, Additional file 1: Fig. S7A, B). Interestingly, minor alterations in the transcript levels of eIF4A1 were noted post-treatment (Additional file 1: Fig. S7C, 8A).

The significant reduction in cellular proliferation coupled with decreased nascent peptide biosynthesis upon compound treatments encouraged us to interrogate the eIF4A-sensitive genes. Several target oncogenes, including cMYC (Wilmore et al. 2021), MCL1 (Wilmore et al. 2021), and CARD11 (Steinhardt et al. 2014), are well-established eIF4A-dependent oncogenes. Furthermore, genes like CDK7 (Zhao et al. 2019), NRF2 (Sanghvi et al. 2021), and PARP1 (Parvin et al. 2019) are established chemoresistance markers for various therapies in B-cell lymphoma. As expected, the expression of cMYC, MCL1, and CARD11 was noted to be sensitive to eIF4A activity (Fig. 6C, 6D, Additional file 1: Fig. S7D, E). Surprisingly, emerging chemo-resistant markers, CDK7, PARP1, and NRF2, also decreased dose-dependent (Fig. 6C, D, Additional file 1: Fig. S7D, E). Unlike previous studies (Sanghvi et al. 2021; Peters et al. 2018), we did not observe any significant changes in the protein levels of Cyclin E and BCL2 upon eIF4A inhibition (Fig. 6C, D, Additional file 1: Fig. S7D, E). Furthermore, the mRNA levels of all the above-mentioned oncogenes were minimally modified (Additional file 1: Fig. S7D, S8B). To determine the relative importance of the eIF4A inhibitor in the translation of different mRNAs, we investigated the translational efficiency of most of the mRNAs studied herein (see methodology for details). As shown in Fig. 6E, a heat map summarizes the protein/transcript ratio revealed a population of genes with substantially diminished translation upon the compound treatment (Additional file 1: Fig. S7G).

Given that eIF4A inhibitors demonstrated a statistically significant reduction in cell proliferation in DLBCL subtypes as well as depletion of critical oncogenes, we next performed a colony formation assay with a panel of DLBCL and lymphoblastoid cells. We selected seven different cell lines; five are of DLBCL [ABC (Hagner et al. 2010) or GCB (Truitt and Ruggero 2016)] origin, while the other two are non-malignant cell lines (lymphoblastoid, GMO). In agreement with our previous data, we observed a significant dose-dependent decrease in the number of colonies formed. Notably, RBF 197 displayed minimal effect on colony formation in GMO cell types, while RBF 208 reduced colony formation in GMO cell lines at a higher concentration (Fig. 6F, G, Additional file 1: Fig. S9A, B). These results are consistent with RBF197 and RBF208 being selective eIF4A inhibitors, while the therapeutic window for RBF197 is broader than RBF208.

### Potential RNA clamp mechanism of eIF4A inhibition

**Fig. 7:**
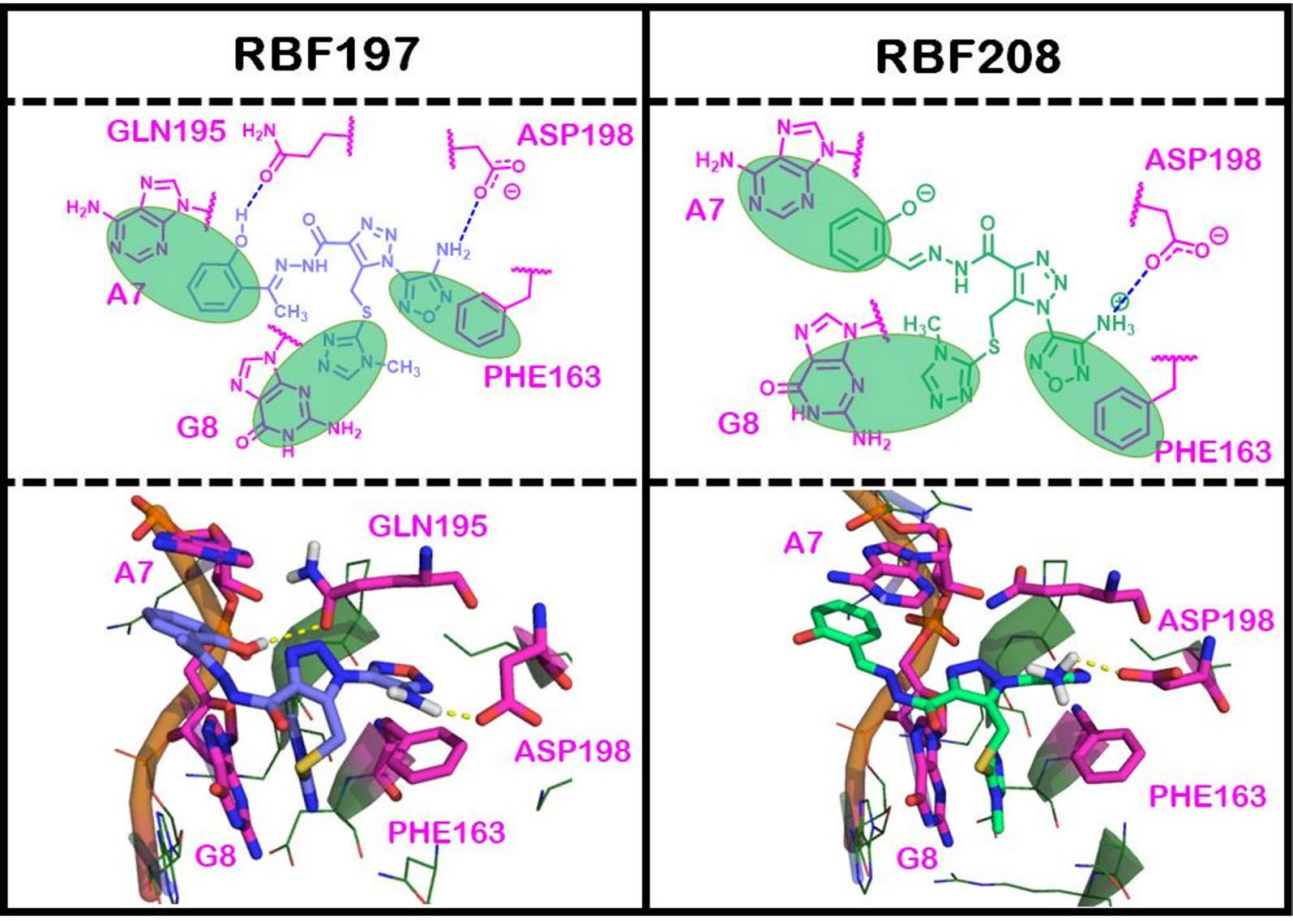
Docking poses of RBF197 and RBF208 in eIF4A:RNA groove. The top two panels show schematic representations of the interactions made between docked poses of RBF197 and RBF208 and their surrounding environments. Green, transparent ovals are used to two-dimensionally represent possible π-π stacking interactions between the ligands and surrounding residues. Dashed lines between the ligands and surrounding residues are used to indicate hydrogen bonding, where the color indicates the donor/acceptor character of the ligand atom (blue = donor; red = acceptor). The lower two panels are high-scoring docked models of RBF197 and RBF208

To account for the increased activities seen for RBF197 and RBF208, we performed more extensive docking studies for these compounds. Not surprisingly, considering their flexibility and the size/shape of the pocket, we obtained a wide variety of high-scoring docked poses for these compounds. Although we obtained many other high-scoring docked poses, we hypothesize that the poses of Fig. 7 are highly probable, as their aromatic ring systems and interactions with GLN195 are consistent with our defined pharmacophore from the original virtual screen, which in turn was based on the RocA-bound crystal structure (Iwasaki et al. 2019) of eIF4A1. Also, the pose shown for RBF197 was the most reasonable since this hit was notably more potent than the others, which we are attributing to a crucial hydrogen bond between the eIF4A1:RNA complex and the protonated, in this case, phenoxide moiety of our scaffold (Figs. 4A and 7).

Our modeling studies suggested that RocA’s and our compounds’ mechanism of action involves trapping and distorting RNA’s bound pose. Indeed, RocA’s crystallized conformation positions itself such that it inserts between the A7 and G8 bases and binds on top of the bound RNA (Fig. 2A). From the results of our virtual screen, we believe our hit compounds bind similarly because they, too, have three aromatic rings capable of forming π-π stacking interactions (Figs. 4A and 7). We speculated that such molecules trap the eIF4A: RNA complex. To experimentally confirm this, we employed a functional RNA unwinding assay to measure the activity of human eIF4A1 recombinant protein in the presence or absence of the inhibitors. Here, the RNA stable duplex was formed by annealing 32mer RNA modified with cyanine 5 (Cy5) at its 5′-end and a complementary 9mer modified with a cyanine 3 (Cy3) at the 3′-end (Additional file 1: Fig. S10A). A stable fluorescence was recorded. A 10-molar excess of unlabeled 9mer was added to the reaction to ensure a single turnover of the RNA unwinding. The reaction was started by adding excess ATP in the presence or absence of compounds. An increase in fluorescence readout was observed in the compound-treated samples compared to DMSO (this was considered basal) (Additional file 1: Fig. S10B). To assess whether the compounds stably locked the eIF4A: RNA duplex, we added an additional fluorescent-labeled stable RNA complex and measured the kinetic values. The values were subsequently normalized and converted to percentage inhibition with respect to DMSO control groups. As proposed, the rate of RNA unwinding of the eIF4A: RNA duplex post-compound treatment was drastically reduced, with the IC_50_ value observed to be upper nanomolar range (Additional file 1: Fig. S10C). The assay results represent the potential RNA-eIF4A-inhibitor complex formation which diminishes eIF4A1’s helicase activity.

## Discussion

Translation initiation, particularly eIF4A RNA helicase, is emerging as a privileged chemotherapeutic target as numerous studies associate it with the rate of protein biosynthesis, tumor initiation, chemoresistance, cancer stem cell functions and metastasis (Fabbri et al. 2021; Park et al. 2019; Lee et al. 2021; Chan et al. 2019). While our current and previous studies (Raza et al. 2015; Andreou and Klostermeier 2013; Liang et al. 2014; Kapadia et al. 2018) support the concept that eIF4A is critical in lymphomagenesis, the clinical utility of selective eIF4A inhibitors has been limited to date. Several potent small molecules have been identified, including natural molecules like rocaglates and elatol, demonstrating potent anticancer activity both in vitro and in vivo. However, none of these compounds has found success in the clinic (Naineni et al. 2020; Chu et al. 2019; Peters et al. 2018; Chu and Pelletier 2018). An important exception is eFT226, a promising candidate undergoing Phase I clinical trials with the data still pending (Ernst et al. 2020). Resistance and relapse to frontline therapy in DLBCL still presents a major clinical issue. Therefore, the successful development of eIF4A-selective small molecules inhibitors as a drug target, may open up new options for therapy of this most common adult lymphoma. Significantly, most potent eIF4A inhibitors, including eFT226, exhibit a common rocaglate backbone, raising the question of whether this chemical backbone is associated with the limiting toxicity. Furthermore, RocA is also reported to bind with prohibitin 1 and 2, thus impeding c-Raf induced MAPK/ERK pathways, raising the question about the specificity of this class of small molecules (Chu et al. 2016).

**Fig. 8:**
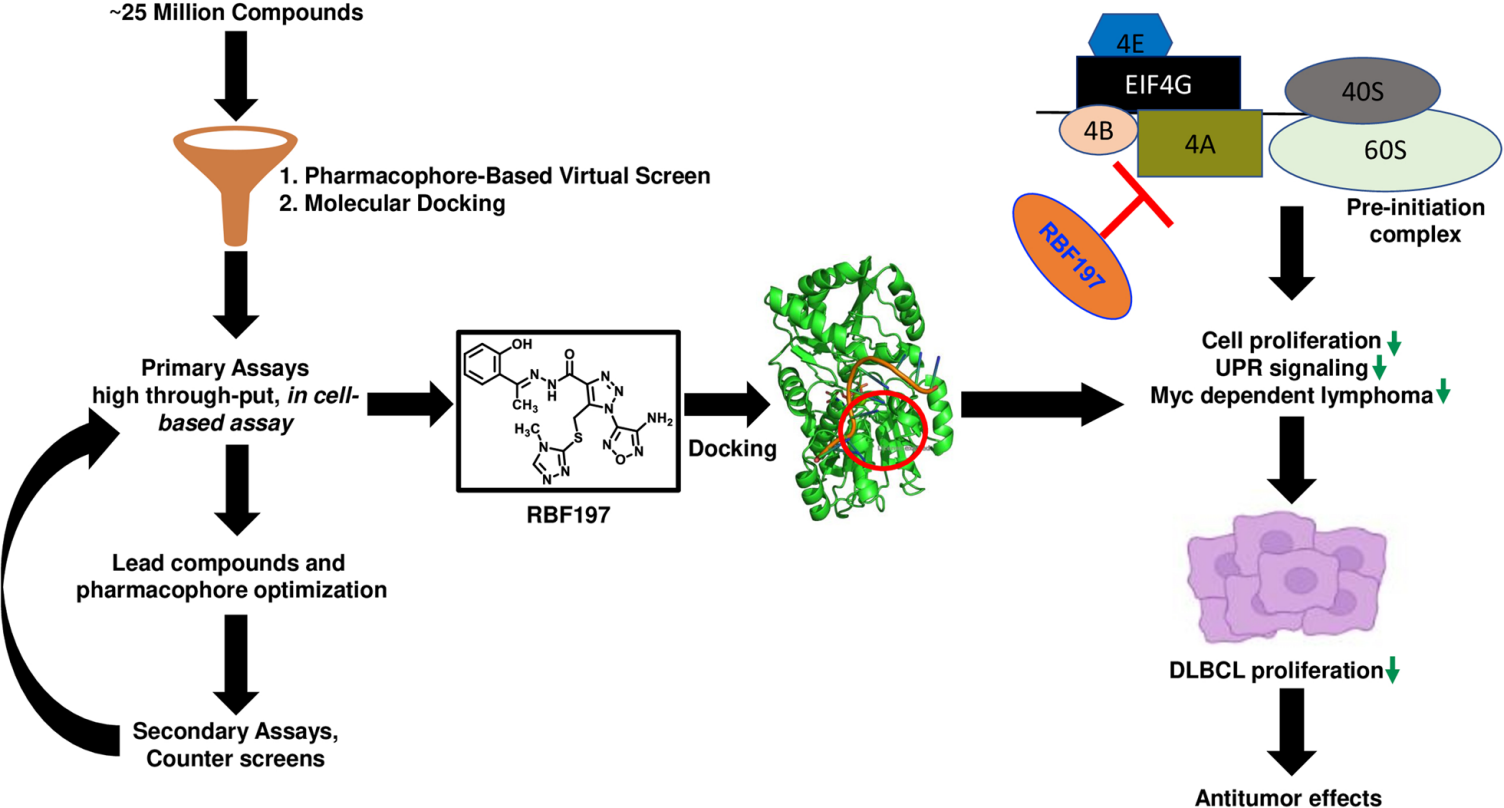
Anticancer effect of RBF197: Schematic summary of identification of novel eIF4A inhibitors and RBF197 targeting eIF4A translational capacity to suppress DLBCL

We utilized the publicly available information about eIF4A1-RocA structure and designed a structure-guided approach to develop RocA-independent potent eIF4A small molecule inhibitors to address these shortcomings. We successfully identified three compounds, RBF197, RBF203, and RBF208, that hamper in vitro eIF4A helicase activity (IC_50_ ≤ 250 pM). Furthermore, through selective replacement of a specific phenoxide moiety, we gained critical mechanistic insights into the mode of action for these small molecules to exert their inhibition. Lastly, we demonstrate that novel eIF4A inhibitors significantly hamper eIF4A-dependent target genes in biologically relevant DLBCL cells (Fig. 8).

Our study began with a survey of the RocA binding site of a RocA::RNA::eIF4A1 co-crystallized complex. Using the HINT force field, we identified several key interactions that we attributed to RocA’s tight binding to the RNA::eIF4A1 complex, including three π-π stacking and two different hydrogen-bonding interactions. These major interactions were utilized as features for a ligand pharmacophore-based virtual screen for novel eIF4A inhibitors. We obtained 1218 hits from our screen, which underwent high-throughput docking in GOLD using our original pharmacophore features as docking constraints. We purchased the top 29 best scoring compounds for primary screening. Based on the previously established screening protocol targeting eIF4A1, we developed a luminometric method for screening eIF4A activity assay by measuring the eIF4A-sensitive 5’UTR driven luciferase readout (Wolfe et al. 2014). This method is simple, sensitive, robust, and in-cell, providing quick and reliable outputs. Comparatively, all the other small molecules targeting eIF4A have been screened using in vitro assays (Naineni et al. 2020; Abdelkrim et al. 2018). Our preliminary screen noted RBF98 impeding 50% luciferase inhibition at 1 nM concentration in the luciferase-based assay while having minimal impact on control assays (Additional file 1: Fig. S3A). Next, we ran it through an in vitro assay, and it observed that the compound inhibits ~ 50% of eIF4A helicase activity around 3 μM. We identified potential new interaction sites, like ASP198 and the known RocA binding sites of PHE163. This is important to note because our docking studies suggest that our most potent compounds from screen utilize similar features as rocaglates for binding and additional ones that may improve its selectivity for eIF4A1 and drug-like properties. The bountiful information we have obtained from our docking studies will further guide the design of new, more potent compounds.

Using these insights, we utilized a rational approach and searched for procurable chemical mimetics of RBF98 and identified three potent small molecule inhibitors: RBF197, RBF203, and RBF208. All three hits showed a remarkable selective decrease in luciferase assays. Notably, biochemical activity assays demonstrated compounds that are active at picomolar concentrations, which to our knowledge is the first report of eIF4A1 inhibitory activity at this potency. More importantly, all the three novel molecules displayed robust inhibition in cellular proliferation of DLBCLs with EC_50_ ranging in lower micromolar concentrations.

We next extended our study to delineate the mechanistic profiling of eIF4A-dependent transcripts in DLBCL using the MYC/BCL2 DLBCL cell line [RC (Pham et al. 2015)] and the ABC-DLBCL cell line [OCI-LY3 (Wenzel et al. 2013)]. As anticipated, the compounds were effective in blocking translational output in DLBCL (Kapadia et al. 2018). In fact, RBF197 and RBF208 showed a dose-dependent decrease in eIF4A-dependent oncogenes [cMYC (Wilmore et al. 2021), MCL1 (Wenzel et al. 2013), and CARD11 (Steinhardt et al. 2014)]. NRF2 is a redox master regulator induced by oncogenic KRAS regulating the transcriptional program of specific translational factors for efficient protein synthesis (Chio et al. 2016). Further, a recent report indicates that NRF2 activation, an emerging prognostic indicator in DLBCL (Yi et al. 2018), confers resistance to silvestrol analog in cancer therapy (Chio et al. 2016). In contrast, treatment with novel identified eIF4A inhibitors, we noted a dose-dependent decrease of NRF2 at the protein levels. Recent evidence suggests that intramolecular KRas-NRF2 axis are involved in stress granules formation, which are emerging as a key indicator of chemoresistance (Mukhopadhyay et al. 2020). Given that the cancer cells are exposed to adverse conditions both in the tumor micromovement and during chemotherapy, stress granules utilizes the post transcriptional control mechanism to re-program gene expression for enhancing cellular survivability (Zhan et al. 2020; Anderson et al. 2015). Importantly, enhanced activity of eIF4A has been reported to reduce RNA condensation and thus limiting stress granules formation under favorable cellular proliferative conditions (Tauber et al. 2020). Given our observation that eIF4A inhibitors limit the expression of NRF2, future studies will focus on understanding the impact of RBF197/203 on RNA condensation and stress granules activities. Similarly, CDK7, a critical cell cycle modulator deregulated in cMYC and BCL6 dependent DLBCL(Lacrima et al. 2007), was also depleted upon the compound treatment. Likewise, PARP1, a DNA binding protein associated with DNA damage repair that confers resistance to genotoxic compounds routinely used as chemotherapeutic agents(Hu et al. 2018), was also noted to decline in DLBCL cells. Along with genotoxic stress, there is a growing appreciation for understanding the metabolic phenotypes imposed by harsh cancerous conditions for exploring personalized medicine approach to target metabolism in cancer (Martinez-Reyes and Chandel 2021; Mukhopadhyay et al. 2021). Interestingly, in the last decade, several independent authors reported that eIF4A activity is associated with translational adaptation particularly in the context of metabolic plasticity (Castelli et al. 2011; Tsokanos et al. 2016). Thus, it will be very informative to explore mechanistically the role of novel eIF4A inhibitors in regulating the cancer metabolism.

One of the major limitations of the previously reported eIF4A inhibitors like (Peters et al. 2018) was unintended cytotoxicity under cellular studies. Thus, to define the therapeutic window, we performed the colony formation assays using malignant DLBCL cells and non-malignant transformed lymphoblastoid (GMO cell lines) cells. Surprisingly, RBF197 has a therapeutic edge over the counterpart RBF208 by showing the least toxic effect on the GMO cell colonies while inhibiting DLBCL colonies in a dose-dependent manner.

In silico analysis indicated the presence of a crucial hydrogen bond between the GLN195 of eIF4A1:RNA complex and the phenol moiety protonated, in this case, phenoxide moiety of our most active hit scaffold RBF197 (Fig. 7), which potentially explains its higher potency over our other hits. Notably, this docked pose of RBF197 forms the GLN195-phenol hydrogen bond with the acceptor end of GLN195’s amide, which is different from RocA’s hydrogen bond with the donor end. We also believe our docking suggests that our best hit compounds act by trapping eIF4A1 in an RNA-bound state. To address this, we performed an RNA trap assay to delineate the mechanism of action of the novel pharmacophores. This uncompetitive mechanism means that RBF197 or RBF208 prefers to bind eIF4A when the protein is in the RNA-bound state, which is advantageous, as binding RNA to eIF4A facilitates creating a clamp and leads to the unavailability of the enzyme for the next turnover cycle. This mechanism is more beneficial than the ATP inhibitors, resulting in unwanted toxicity in the cells.

## Conclusion

Further work is required to develop a deeper understanding of this novel small molecule series and to describe in detail the mode of eIF4A inhibition. Additional virtual screening coupled with medicinal chemistry design and synthesis of novel compounds will likely lead to even more efficacious lead compounds. As noted above, we have to date only explored analogues that vary the *m*-phenoxide moiety of RBF98, and significant scope for structure optimization is available in other regions of these molecules. Nevertheless, the compounds reported here are potent and unique structural eIF4A inhibitors forming different sets of interactions than the previously known inhibitors. These inhibitors have potential pharmacological relevance and present a valuable therapeutic opportunity.

## Supplementary Information

**Additional file 1: Figure S1.** eIF4A1 expression in molecular subtypes of DLBCL in microarray datasets. A) eIF4A1 expression was found to be significantly (***p < 0.001) higher in ABC-DLBCL subtypes compared to GCB and UN-DLBCL in the microarray dataset GSE10846. B) A similar trend was observed in the GSE87371 dataset p = 0.203. **Figure S2.** Assessment of eIF4A1 specific luciferase assay in Hek293T/17 stables. A) Design of luciferase construct with 5’UTR of eIF4A1 G-quadruplex sequence with the β-actin promoter, negative controls, blank with scrambled sequence and empty test construct. B) eIF4A-3X-Luciferase expressing 293 T cells were transfected with shRNA against eIF4A1 and eIF4B followed by luciferase readout. Statistics were performed using Dunnett's Test. A significant decrease was observed in relative luciferase units in eIF4A1 shRNA groups. ****p < 0.0001 vs non-transfected cells C) Various dose treatments of silvestrol in eIF4A1-3X, blank and empty luciferase Hek293T/17 stables. Percentage inhibition in the reduction of luciferase was calculated relative to DMSO control. The IC50 value was observed to be 10 nM. Statistical analysis was performed using one-way ANOVA followed by Bonferroni’s correction analysis. ap < 0.05; cp < 0.001, dp < 0.0001 vs DMSO control groups, αp < 0.05, βp < 0.001, ¥p < 0.0001 vs 1 μM or 10 μM treatment groups. **Figure S3.** RBF98 activity in luciferase and biochemical assays A) Percentage inhibition of luciferase activity on treatment with RBF98 at 0.1, 1, and 10 µM concentrations in empty/blank luciferase HEK293T/17 stable cell lines. Statistical analysis was performed using one-way ANOVA followed by Bonferroni’s correction analysis. ap < 0.05; cp < 0.001, dp < 0.0001 vs DMSO control groups, αp < 0.05, βp < 0.001, ¥p < 0.0001 vs 10 μM treatment groups. B) eIF4A1 titration and measurement of phosphate release with 50 μM ATP and 100 ng/ml of yeast RNA. C) ATP titration to select linear range concentration in presence of 20 ng of eIF4A1 and 100 ng/ml yeast RNA. D) Dose-dependent percentage inhibition of human eIF4A1 in-vitro activity on the treatment of RBF98, compared to DMSO control using inorganic phosphate release assay (SensoLyte kit). IC50 values observed were observed to be 3 µM. **Figure S4.** RBF98 reduces proliferation and overall translation in DLBCL A) For proliferation assay, Farage (GCB) origin was seeded at a density of 10,000 and treated with 0.5 and 1 µM of RBF98 for up to 72 h. The cell viability was measured at different time points using the trypan blue method. Silvestrol treatment was done at 50 nM as a positive control group. Viability was observed to be decreasing with increasing time in comparison to DMSO control (ap < 0.05; cp < 0.001, dp < 0.0001). B) Effect of RBF98 on DLBCL colony formation. The total number of colonies grown in Toledo (malignant), GMO1528, and GMO13604 (non-malignant) cells on treatment with 0.5 and 1 mM of RBF98. Statistical analysis was performed using one-way ANOVA followed by Bonferroni’s correction analysis. ap < 0.05; cp < 0.001, dp < 0.0001 vs DMSO control groups, αp < 0.05, βp < 0.001, ¥p < 0.0001 vs 1 mM treatment groups. C) Puromycin (1 μg/mL) was exposed, post compound treatments for 30 min (SUnSET assay), and cells (Toledo and OCI-LY3) were lysed. Representative immunoblots probed with anti-puromycin, anti-eIF4A1, anti-eIF4E, anti-cMYC, anti-CyclinD1. GAPDH was used as the loading control. **Figure S5. **Percentage inhibition of luciferase activity. On treatment of RBF197, 203, and 208 respectively at 0.1, 1, and 10 µM concentrations A) empty and B) blank luciferase HEK293T/17 stable cell lines. Average inhibition in relative luciferase units was not found to be more than 20% at the given concentrations. Statistical analysis was performed using one-way ANOVA followed by Bonferroni correction analysis. ap < 0.05; cp < 0.001, dp < 0.0001 vs DMSO control groups, αp < 0.05, βp < 0.001, ¥p < 0.0001 vs 10 μM treatment groups. **Figure S6.** RBF197 attenuates DLBCL growth. A) Concentration–response curves of RBF197 in SUDHL4, OCI-Ly3, DS, and RC cell lines. Linear regression fit of the inhibition values was plotted using Graph pad prism and EC50 values were calculated. The EC50 values were observed to be less than 10 µM. **Figure S7.** RBF197 and 208 decrease overall translation and eIF4A1 dependent pathway proteins in ABC-DLBCL OCI-Ly3 cell line. A) Representative immunoblots probed with anti-puromycin, anti-eIF4A1, and anti-eIF4E. B, D) Relative fold change in protein levels with on treatment of RBF 197 (B) or RBF208 (D) at 1 and 3 µM concentrations with respect to the DMSO control. GAPDH was used as a loading control C, E) qRT-PCR analysis for the expression of defined genes in RC with the treatment of RBF 197 (C) or RBF208 (E) at 1 and 3 µM. 36B4 was used as a housekeeping gene. Results were normalized with DMSO controls and expressed as mean ± SD (n = 3). F) Representative immunoblots of indicated antibodies on treatment with RBF197 and 208 at 1 and 3 µM concentrations respectively. Vinculin was used as a loading control. G,I) Relative fold change in protein levels of the above-mentioned proteins with on treatment of RBF197 (G) and RBF208 (I) respectively. H, J) Relative mRNA expression data for defined genes with the treatment of RBF197 (H) and RBF208 (J) respectively. 36B4 was used as a housekeeping gene. Results were normalized with DMSO controls and expressed as mean ± SD (n = 3). Statistical analysis was performed using one-way ANOVA followed by Bonferroni’s correction analysis. ap < 0.05; cp < 0.001, dp < 0.0001 vs DMSO control groups, αp < 0.05, βp < 0.001, ¥p < 0.0001 vs 3 μM treatment groups. K) Heat map of translation efficiency values for RBF197 and RBF208 in OCI-Ly3 cell line. **Figure S8.** RBF197 and RBF208 decreases overall translation and eIF4A1 dependent pathway proteins in double-hit lymphoma. A) qRT-PCR analysis for expression of defined genes in RC following treatment of RBF197 or RBF208 at 1 and 3 µM. B) Relative mRNA expression data for defined genes with the treatment of RBF197 and RBF208 respectively. **Figure S9. **Effect of RBF197 and RBF208 on DLBCL colony formation. A, B) The total number of colonies grown in Farage, SUDHL4, DS, OCI-Ly3 (malignant), GMO17220B (non-malignant) cell lines upon treatment with 1 and 3 µM of RBF197 (A) and RBF208 (B) respectively. Statistical analysis was performed using one-way ANOVA followed by Bonferroni’s correction analysis. ap < 0.05; cp < 0.001, dp < 0.0001 vs DMSO control groups, αp < 0.05, βp < 0.001, ¥p < 0.0001 vs 3 μM treatment groups. **Figure S10. **RBF 197 and RBF208 inhibit human eIF4A1 by RNA clamp mechanism. A) Schematic depiction of the fluorescent duplex unwinding assay. A 32-mer RNA strand is modified on its 5′ end with cyanine 5 (Cy5) and annealed to a complimentary loading strand that is modified on its 3′ end 9-mer with cyanine 3 (Cy3). The 9-mer RNA strand released upon unwinding was trapped by an unlabeled 9-mer DNA oligonucleotide to give single turnover reactions. B) Four-step protocol, 1. Duplex formation (5 µM) by heating at 960C for 2 min, cooling on ice for 5 min. 2. Read stable fluorescence at 0 min with or without compound in presence of human eIF4A1, unlabeled 9mer, and additional duplex. 3. Addition of ATP and record kinetic reading for 30 min. 4. Post 30 min, again add duplex and read the fluorescence intensity after 3 min C) The difference in the increase in the fluorescence was calculated in the presence and absence of RBF197 and RBF208. Concentration–response curves were plotted using a graph pad prism and IC50 was observed at 0.7 and 0.9 µM respectively.

**Additional file 2: Supplemental Table 1.** Sequence of the primers and probes used. **Supplemental Table 2.** Compounds screened, their corresponding Molport ID and IUPAC names (screen 1). **Supplemental Table 3.** Compounds screened, their corresponding Molport ID and IUPAC names (screen 2). **Supplemental Table 4.** IC50 values of selected RBFs compound in a panel of five DLBCL cell lines using WST-1 assay.

## Data Availability

The data used to support the findings of the present study are available from the corresponding author upon request.

## Abbreviations

eIF4A: Eukaryotic translation initiation factor (eIF) 4A
mRNA: Messenger RNA
RocA: Rocaglamide A
DLBCL: Diffuse large B-cell lymphoma
(5′) m7G: 5-Prime (5′) 7-methylguanosine
5′-UTR: 5′ Untranslated region
HINT: Hydropathic INTeractions
GOLD: Genetic Optimisation for Ligand Docking
SUnSET: Surface sensing of translation
ABC: Activated B-cell
GCB: Germinal center B-cell
UNC: Unclassified B-cell
TCGA: The Cancer Genome Atlas
OS: Overall survival
PFS: Progression-free survival
