# Supplementary figures and images for: Novel eIF4A1 inhibitors with anti‐tumor activity in lymphoma
Source: Mol Med. 2022 Sep 4;28:101. doi: 10.1186/s10020-022-00534-0 (PMC9441068; doi:10.1186/s10020-022-00534-0)

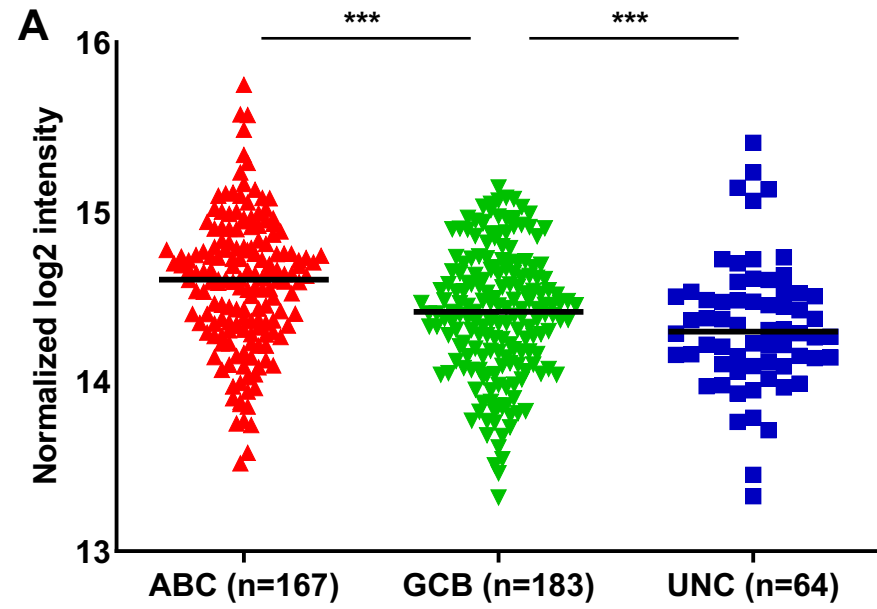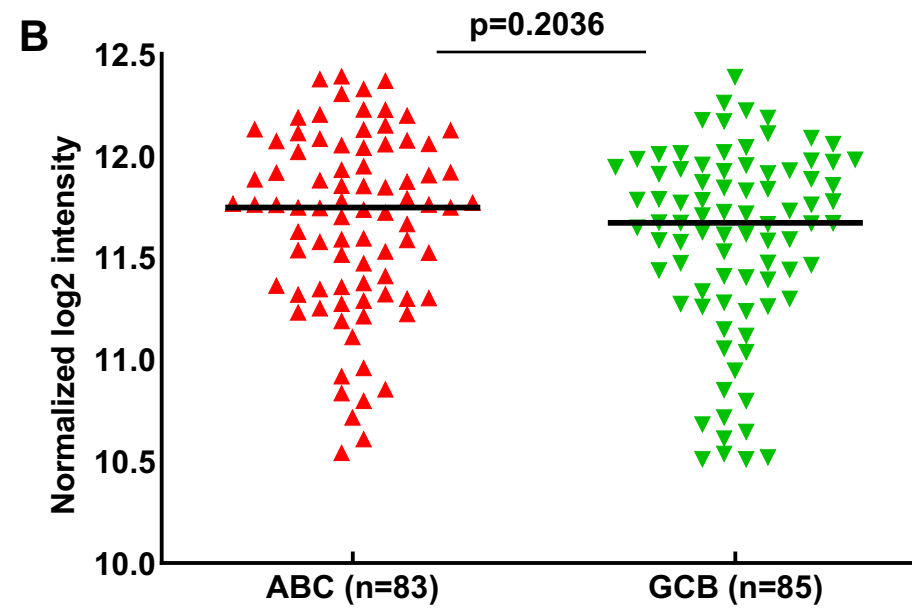

**A**

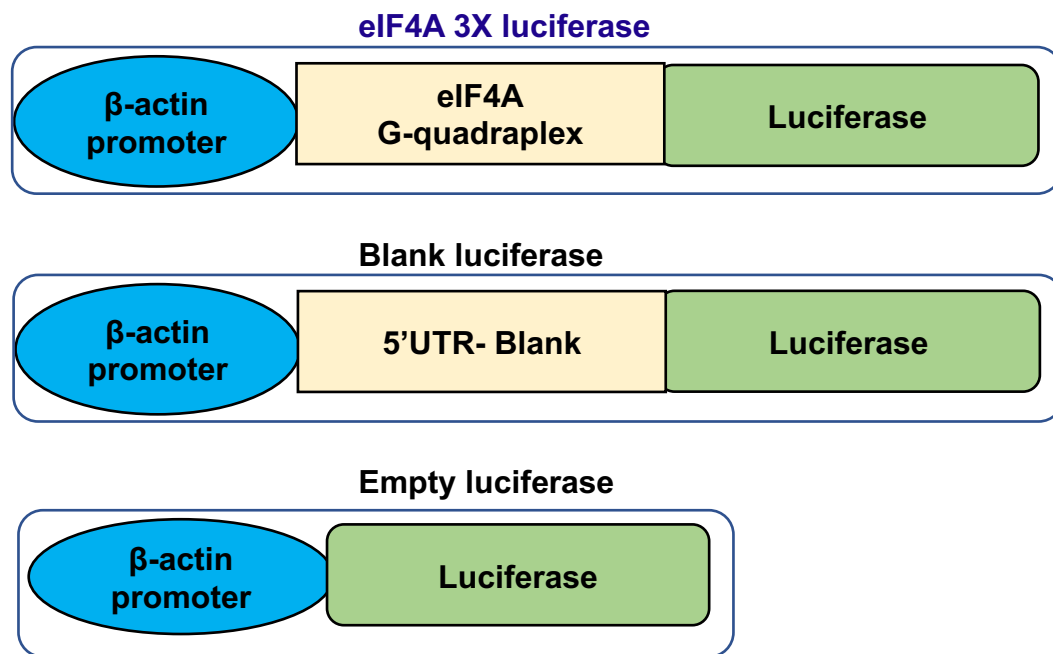

**B**

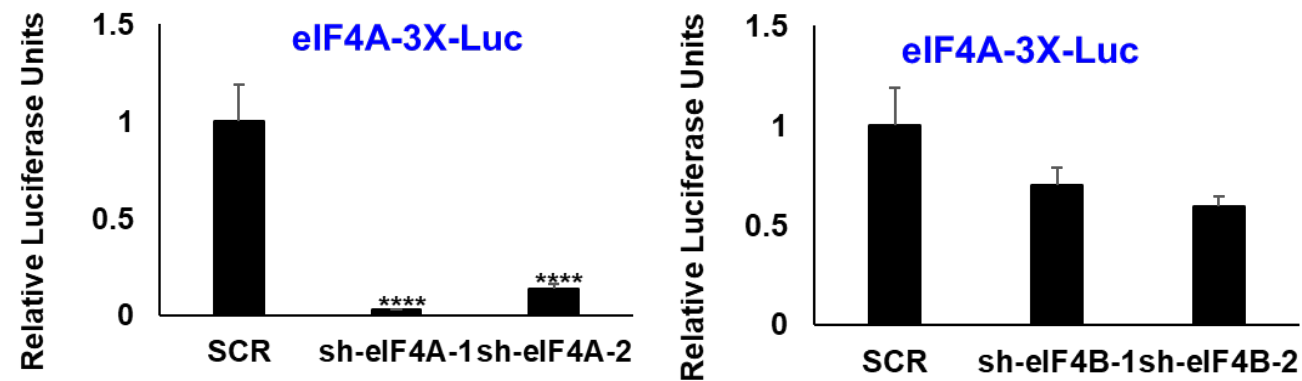

**C**

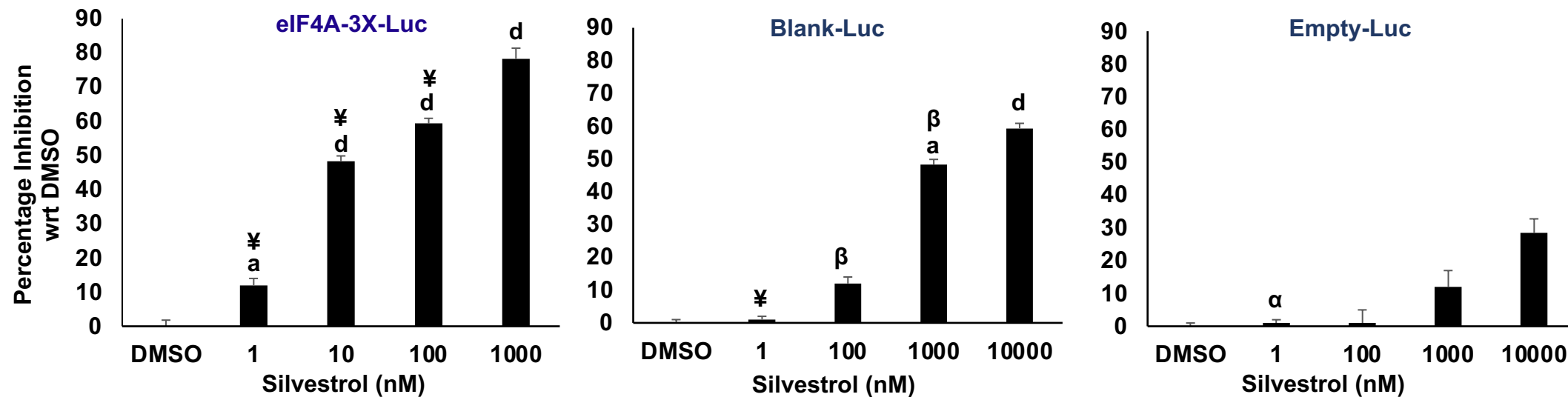

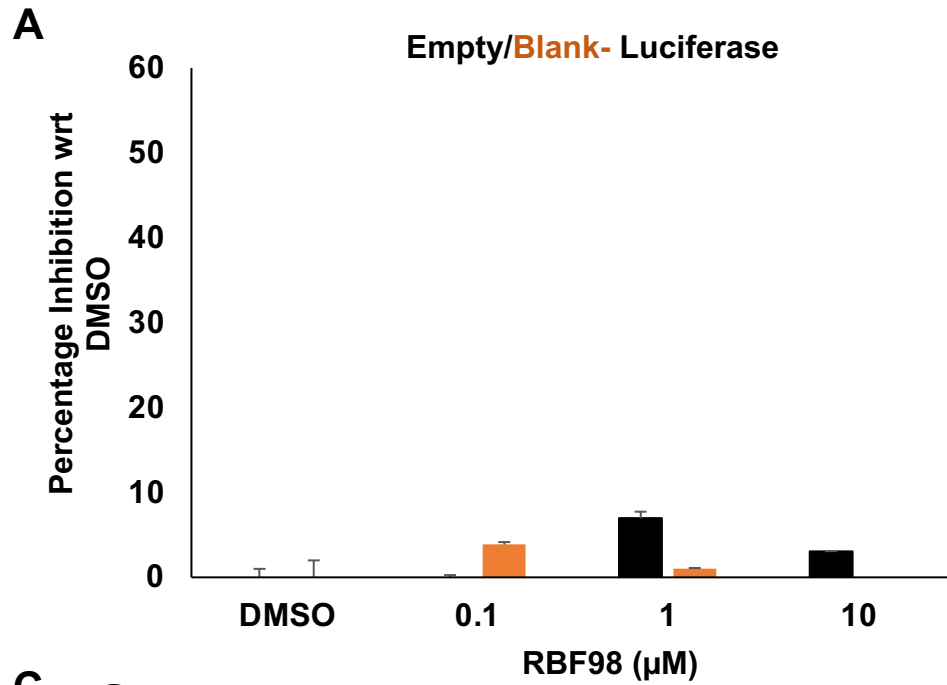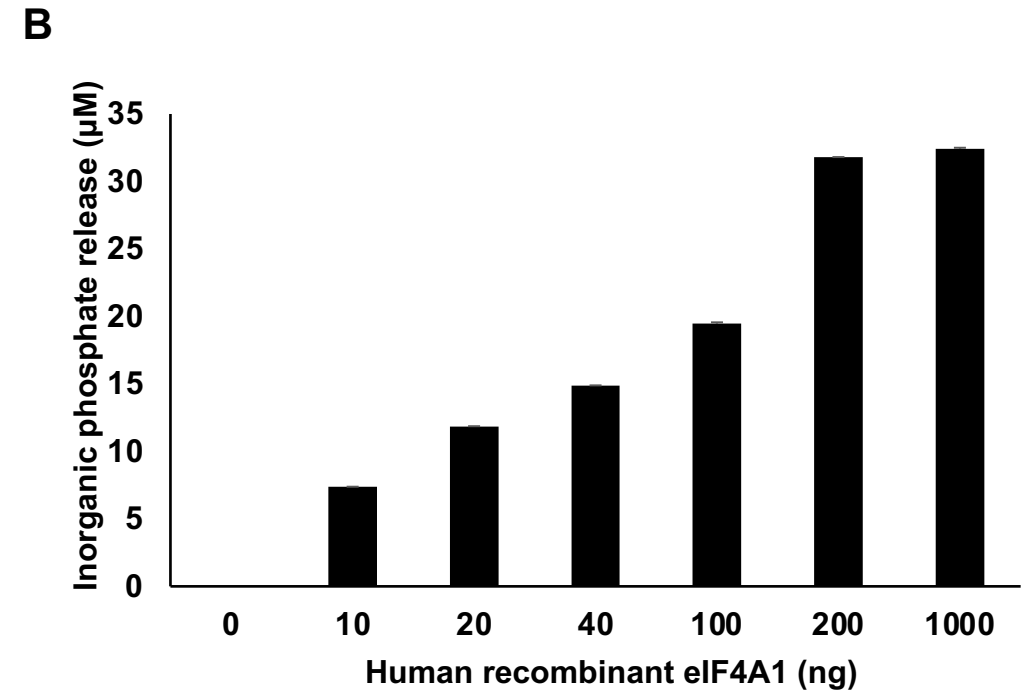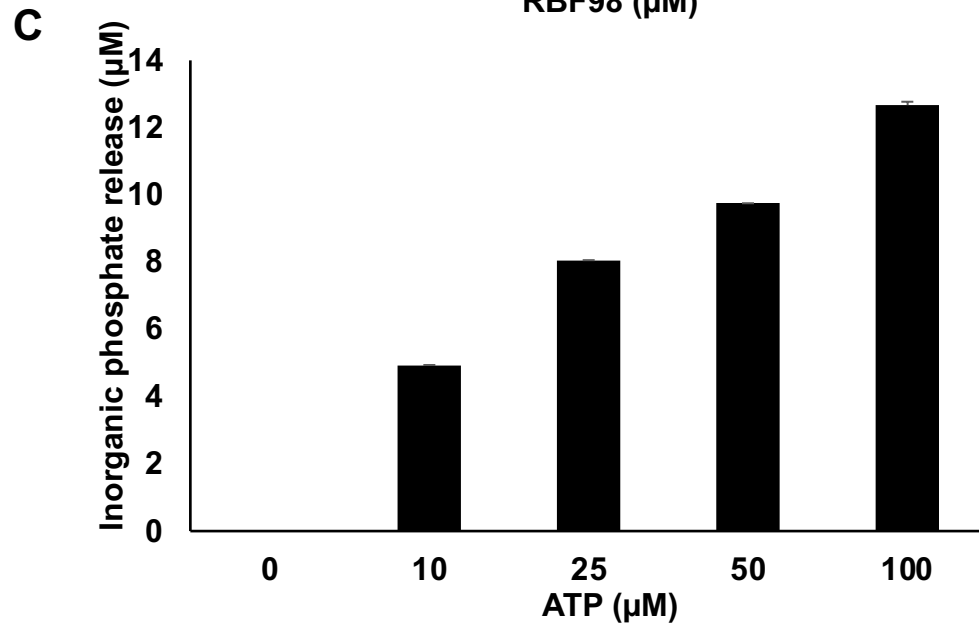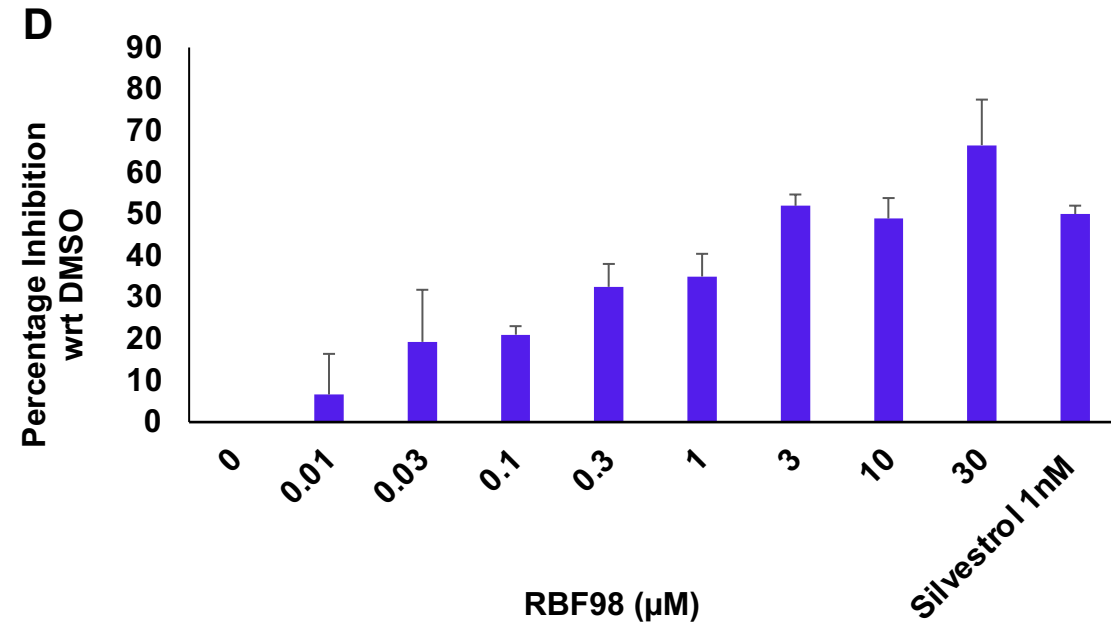

A

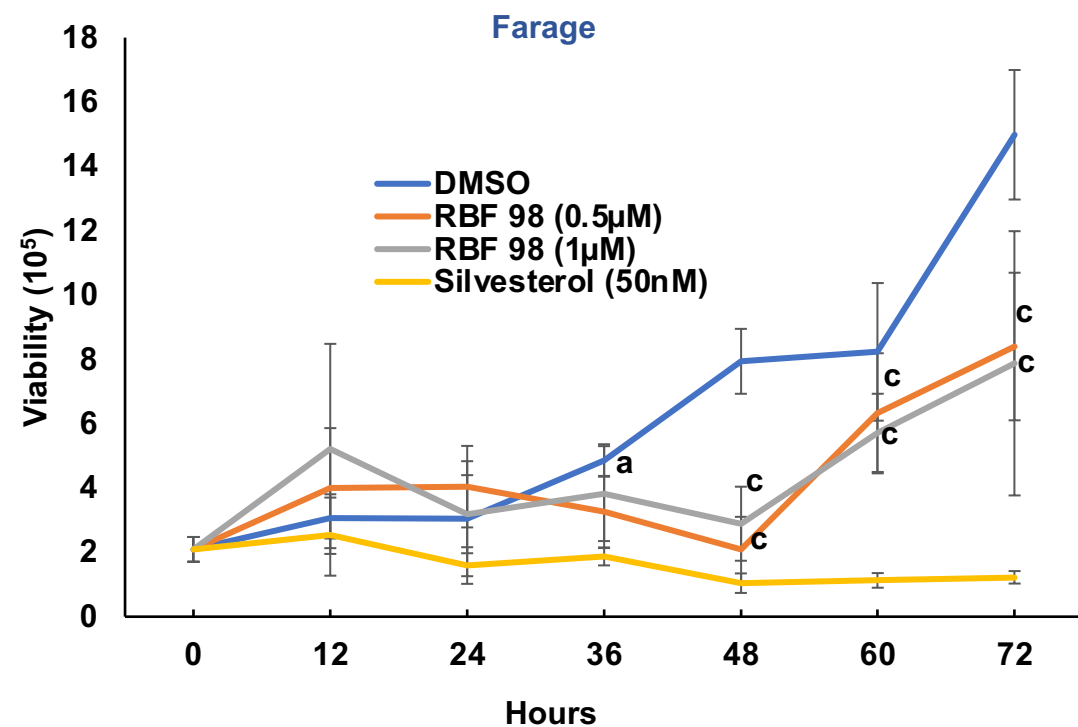

C

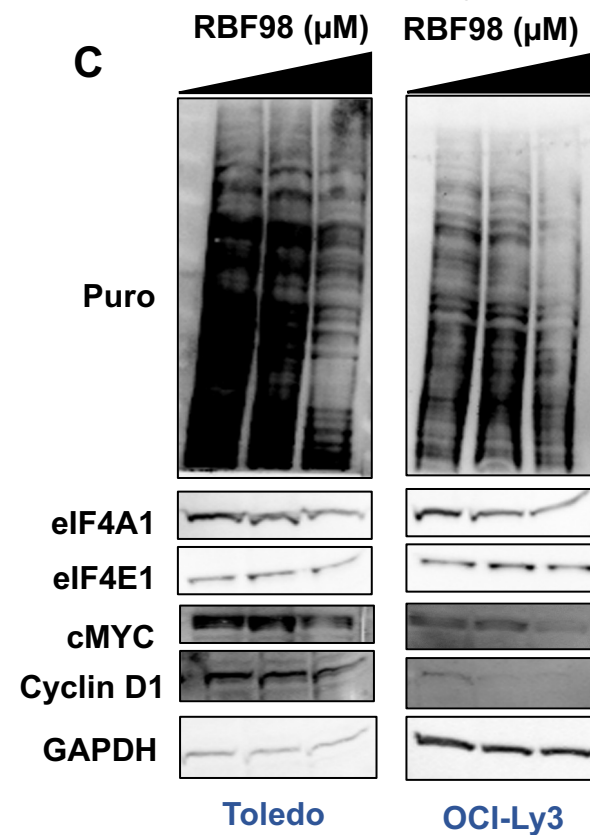

B

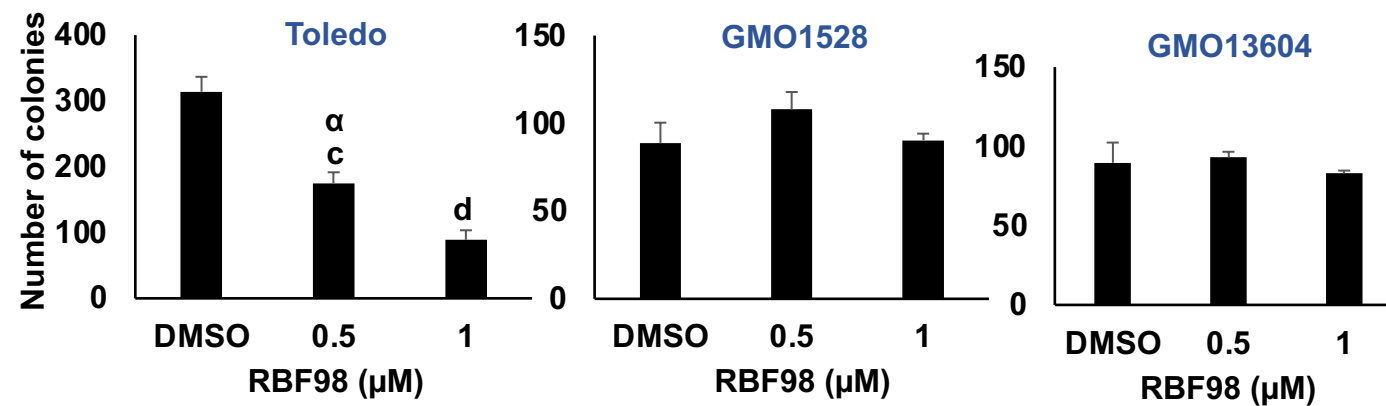

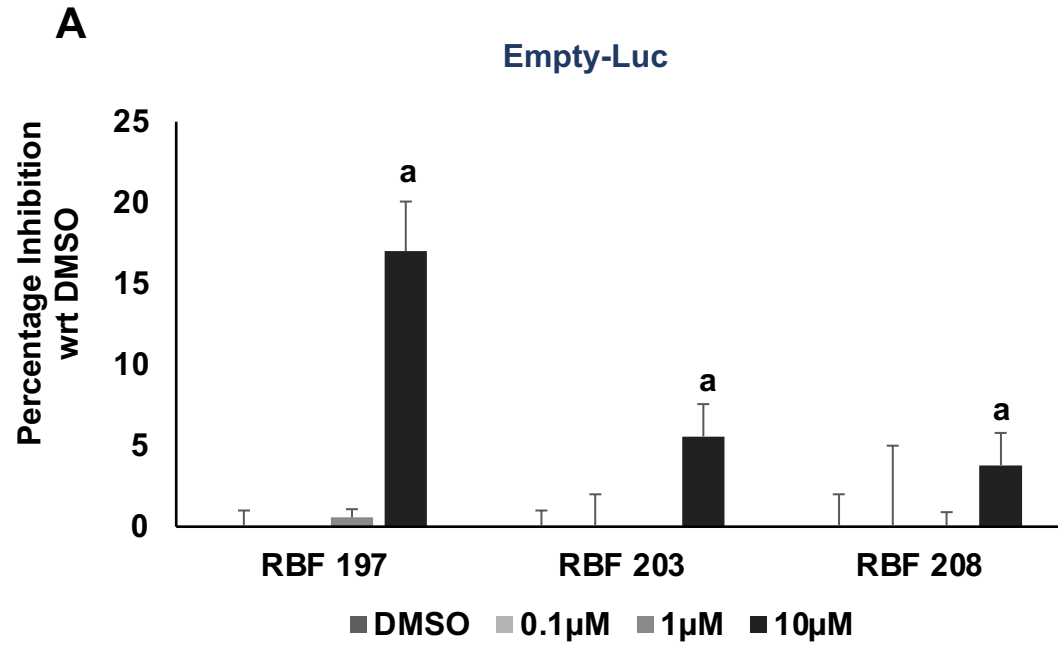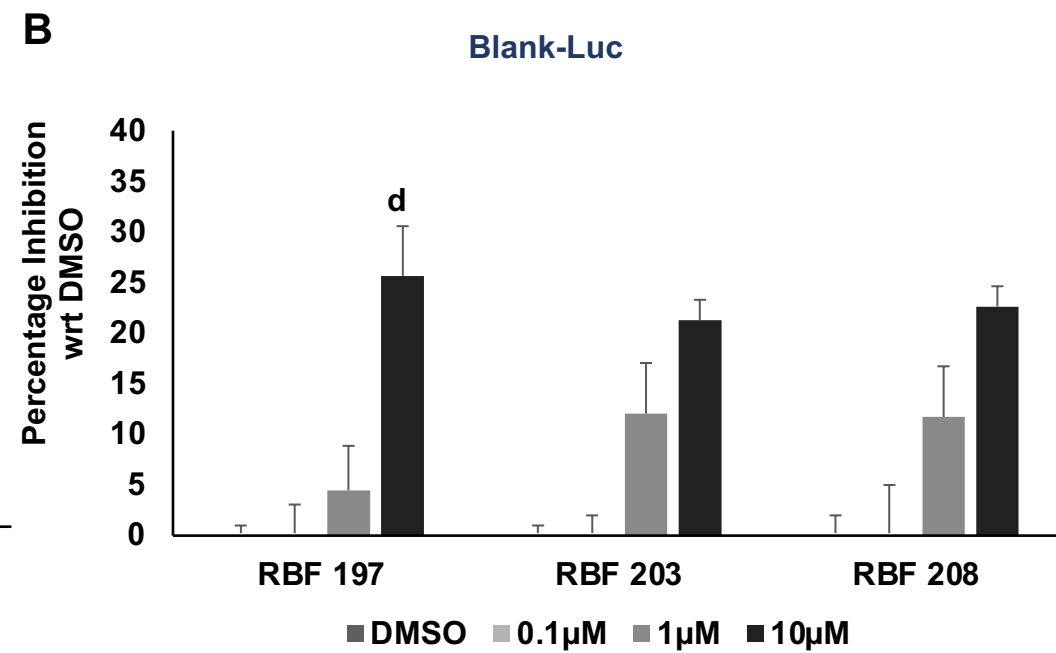

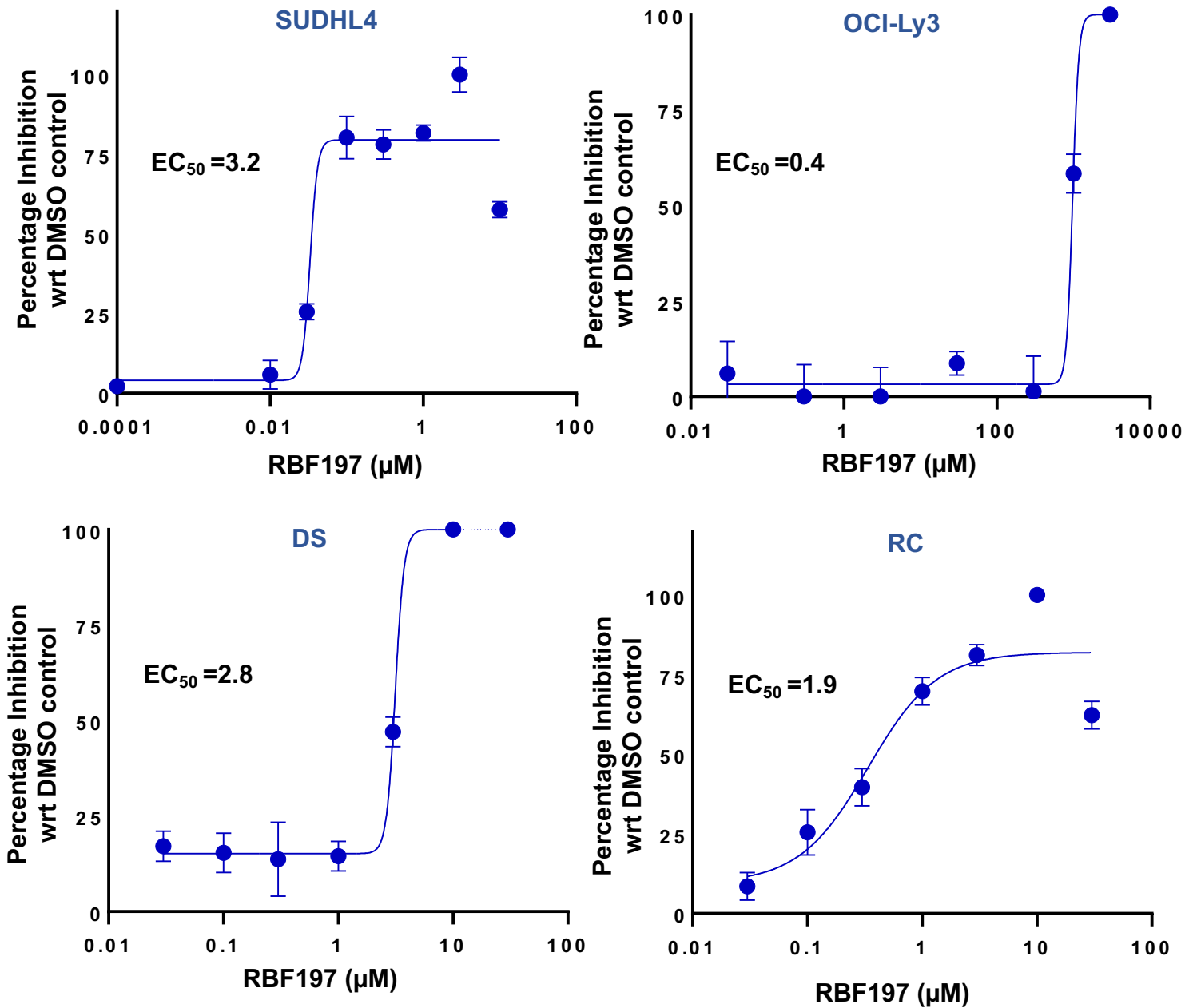

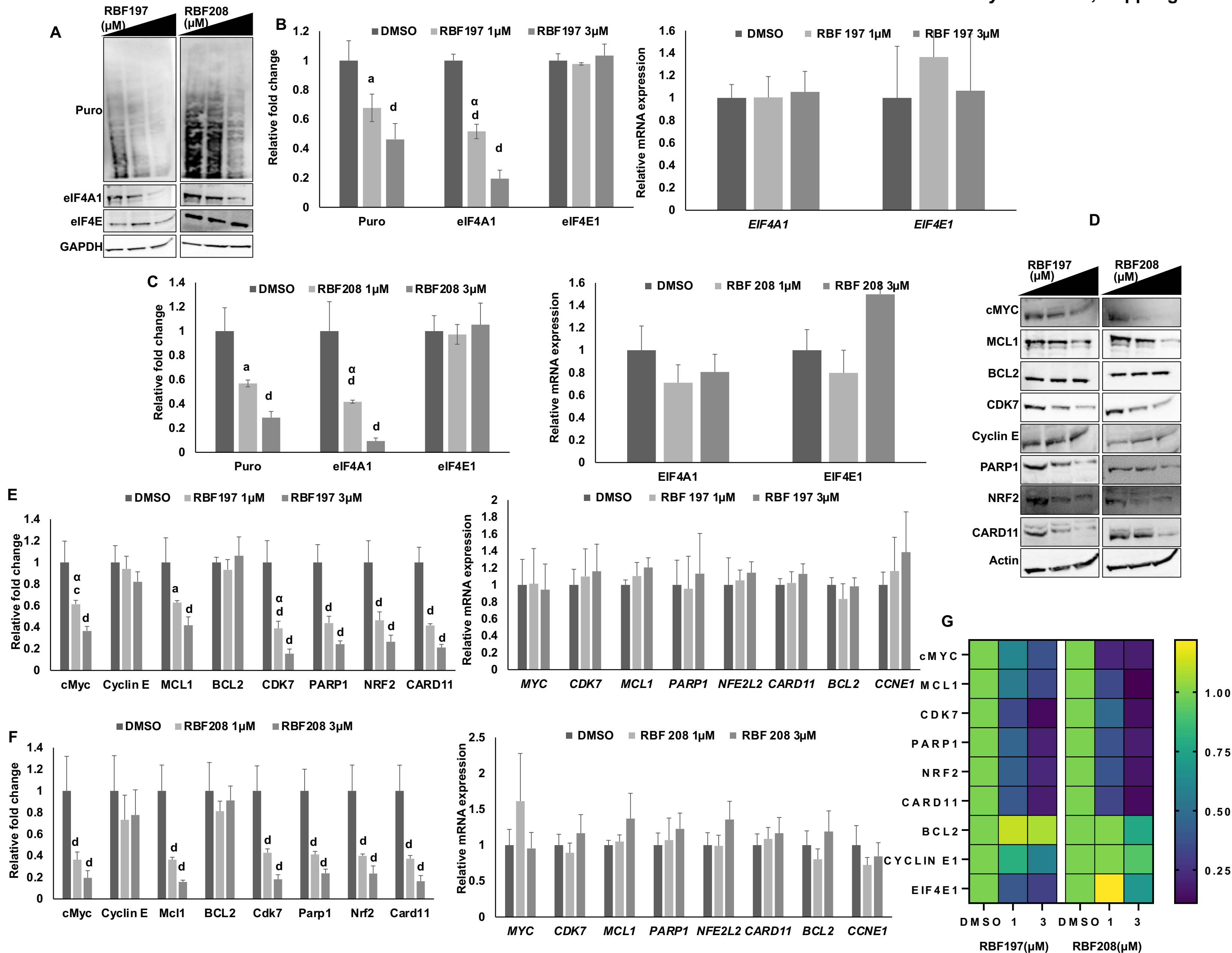

**A**

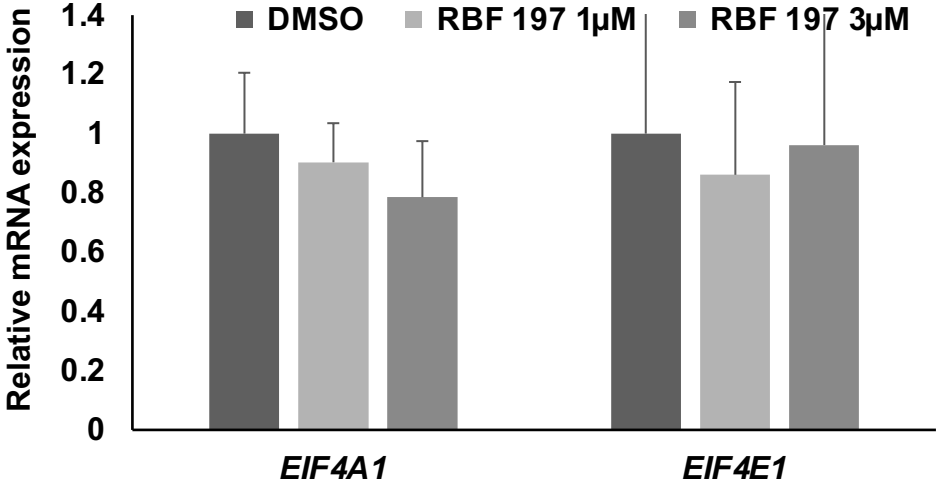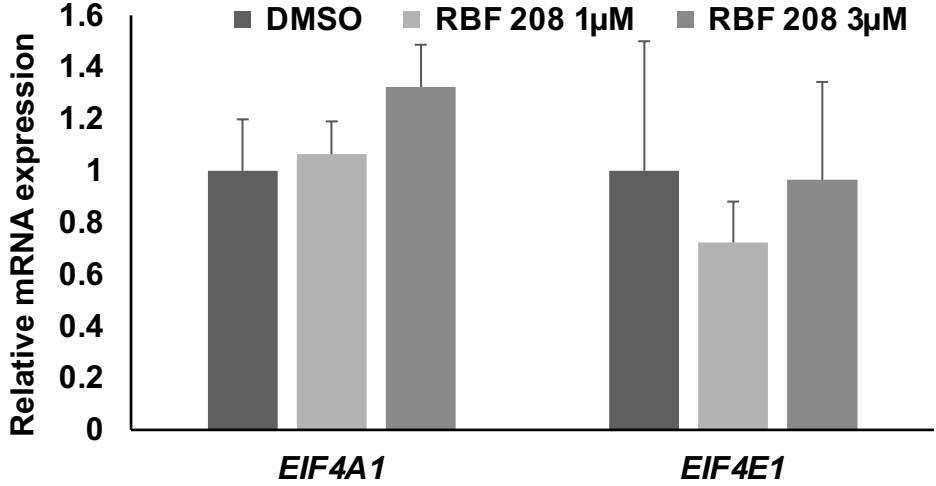

**B**

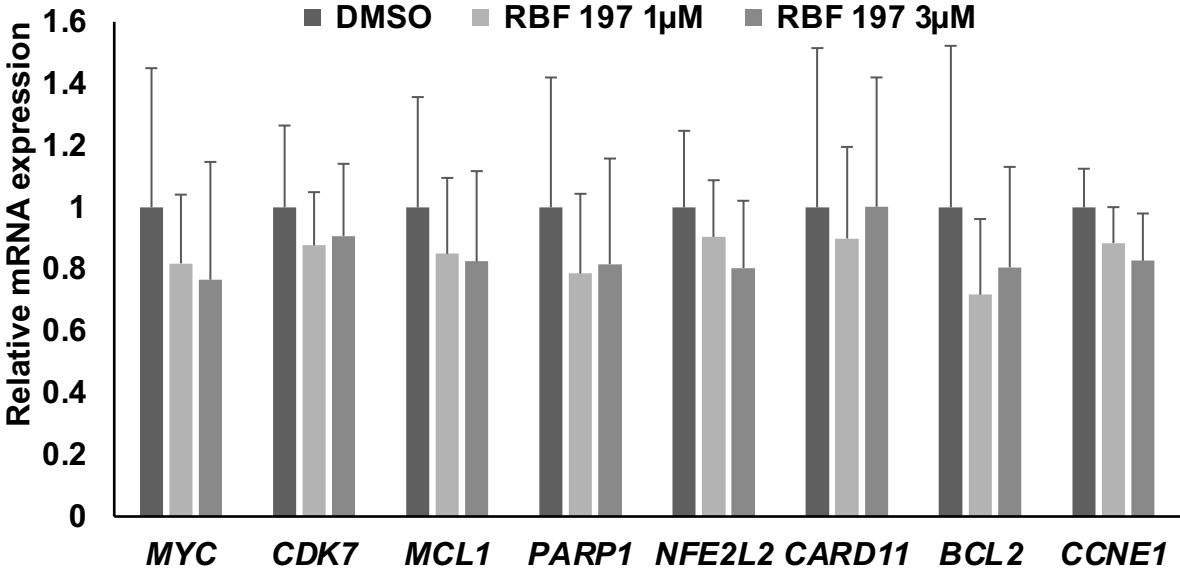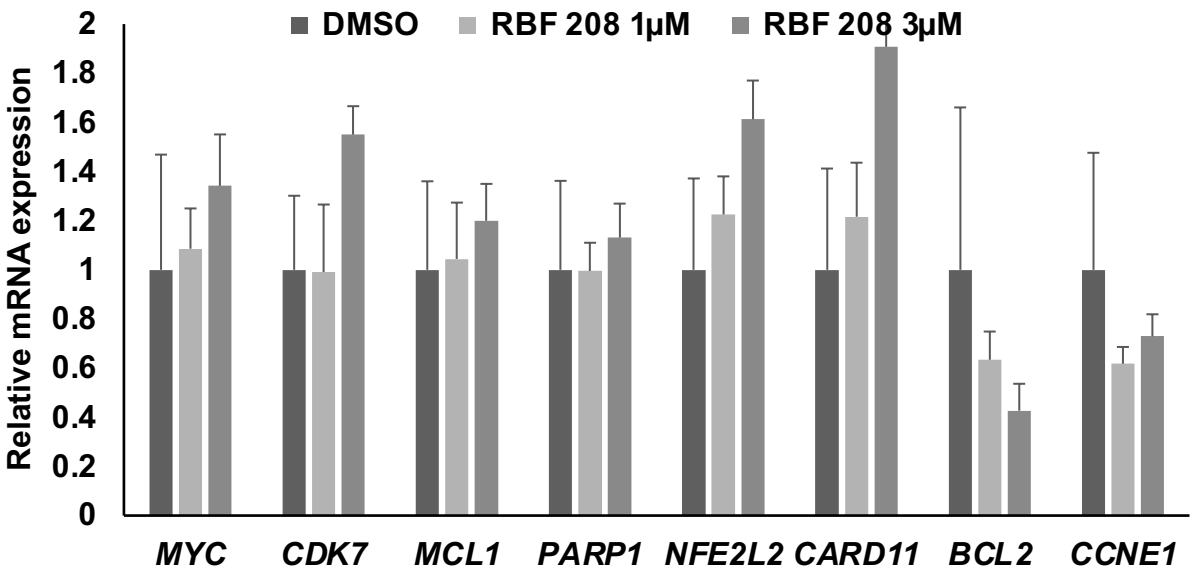

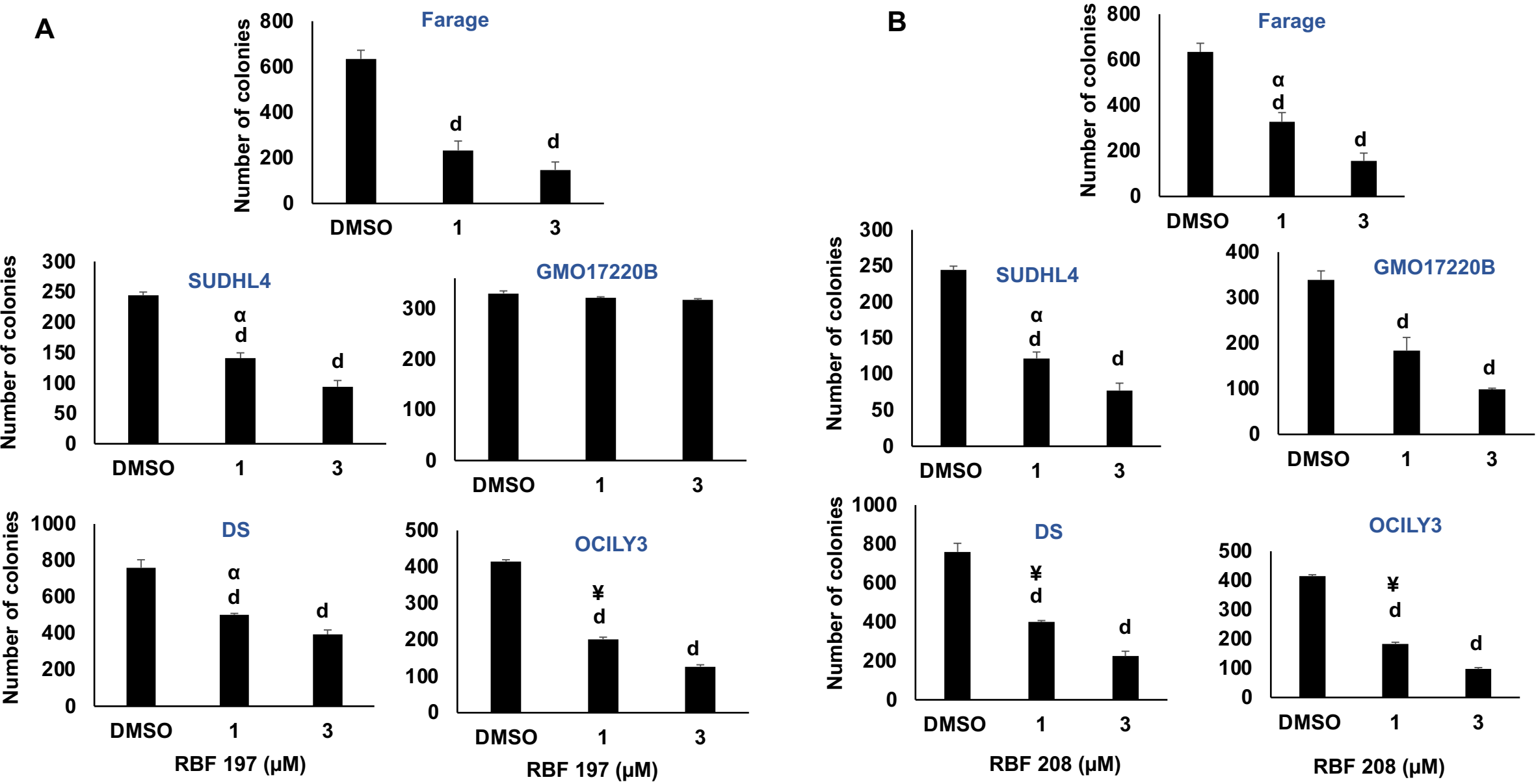

**B**

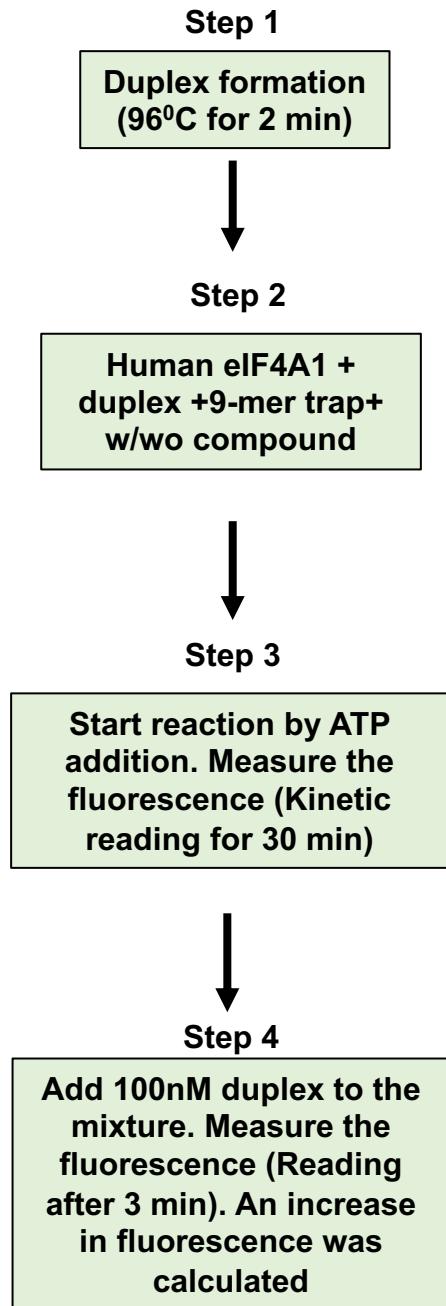

**A**

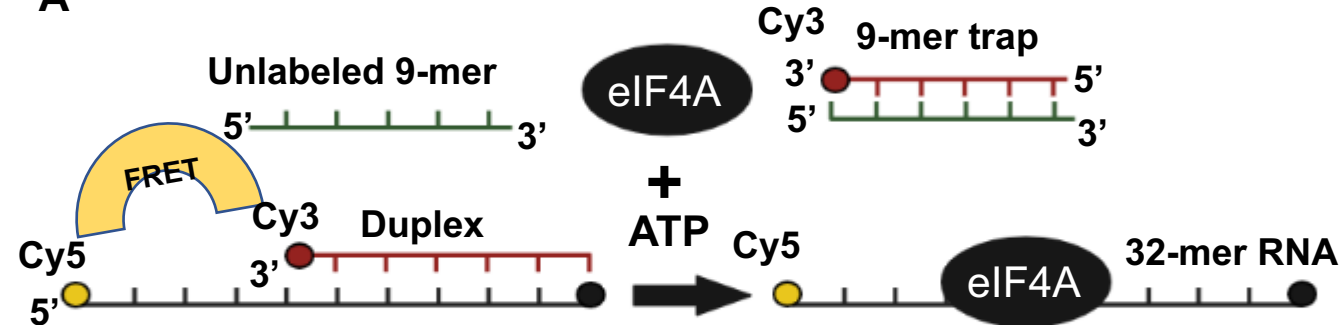

**C**

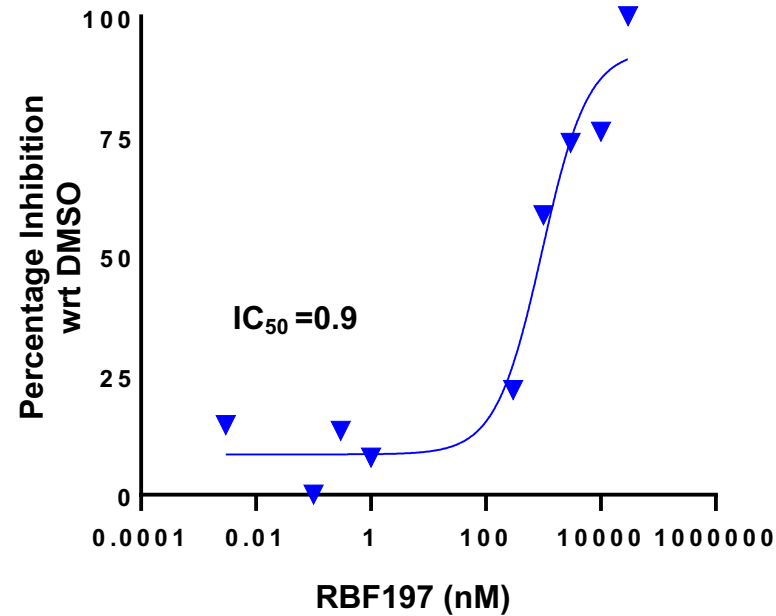

**D**

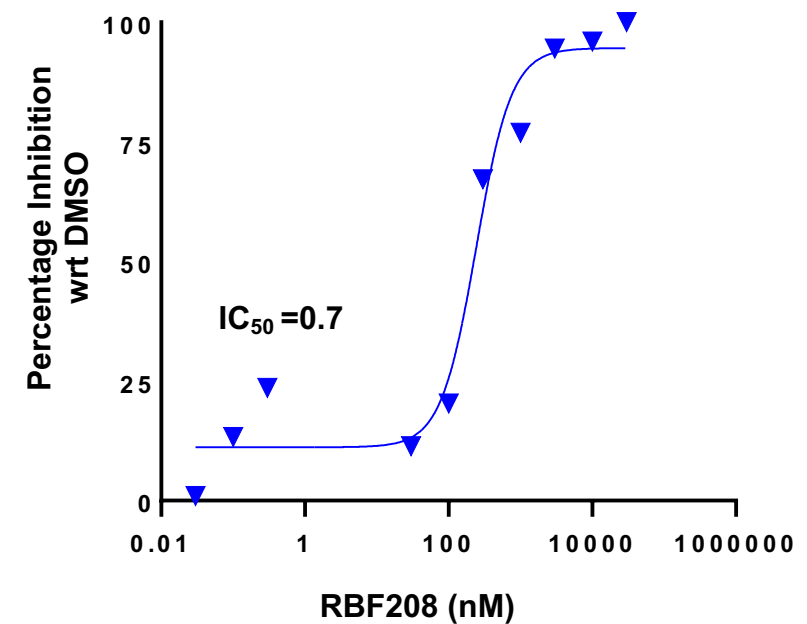

Supplement: Supplementary file 1 — Additional file 1: Figure S1. eIF4A1 expression in molecular subtypes of DLBCL in microarray datasets. A) eIF4A1 expression was found to be significantly (***p < 0.001) higher in ABC-DLBCL subtypes compared to GCB and UN-DLBCL in the microarray dataset GSE10846. B) A similar trend was observed in the GSE87371 dataset p = 0.203. Figure S2. Assessment of eIF4A1 specific luciferase assay in Hek293T/17 stables. A) Design of luciferase construct with 5’UTR of eIF4A1 G-quadruplex sequence with the β-actin promoter, negative controls, blank with scrambled sequence and empty test construct. B) eIF4A-3X-Luciferase expressing 293 T cells were transfected with shRNA against eIF4A1 and eIF4B followed by luciferase readout. Statistics were performed using Dunnett's Test. A significant decrease was observed in relative luciferase units in eIF4A1 shRNA groups. ****p < 0.0001 vs non-transfected cells C) Various dose treatments of silvestrol in eIF4A1-3X, blank and empty luciferase Hek293T/17 stables. Percentage inhibition in the reduction of luciferase was calculated relative to DMSO control. The IC50 value was observed to be 10 nM. Statistical analysis was performed using one-way ANOVA followed by Bonferroni’s correction analysis. ap < 0.05; cp < 0.001, dp < 0.0001 vs DMSO control groups, αp < 0.05, βp < 0.001, ¥p < 0.0001 vs 1 μM or 10 μM treatment groups. Figure S3. RBF98 activity in luciferase and biochemical assays A) Percentage inhibition of luciferase activity on treatment with RBF98 at 0.1, 1, and 10 µM concentrations in empty/blank luciferase HEK293T/17 stable cell lines. Statistical analysis was performed using one-way ANOVA followed by Bonferroni’s correction analysis. ap < 0.05; cp < 0.001, dp < 0.0001 vs DMSO control groups, αp < 0.05, βp < 0.001, ¥p < 0.0001 vs 10 μM treatment groups. B) eIF4A1 titration and measurement of phosphate release with 50 μM ATP and 100 ng/ml of yeast RNA. C) ATP titration to select linear range concentration in presence of 20 ng of eIF4A1 a [file 10020_2022_534_MOESM1_ESM.pdf]
